# A Bi-Level Hybrid Framework for Multi-Target Path Planning of AGV Based on Particle Swarm Optimization and Bidirectional Rapidly Exploring Random Tree

**DOI:** 10.3390/s26134062

**Published:** 2026-06-26

**Authors:** Tursun Mamat, Zhaolong Liu, Qiuju Yang, Abdukeram Dolkun, Longfei Li

**Affiliations:** 1 Engineering Research Center for Intelligent Transportation, School of Transportation and Logistics Engineering, Xinjiang Agricultural University, 311 Nongda East Road, Urumqi 830052, China; 19106416193@163.com (Z.L.); 320232604@stu.xjau.edu.cn (A.D.); 320242634@stu.xjau.edu.cn (L.L.); 2 Key Laboratory of Transportation and Logistics Engineering in Xinjiang, School of Transportation and Logistics Engineering, Xinjiang Agricultural University, 311 Nongda East Road, Urumqi 830052, China; 3 Xinjiang Communications Construction Group Co., Ltd., Urumqi 830016, China; 15999176681@163.com

**Keywords:** AGV, multi-target path planning, Bi-RRT, PSO, adaptive sampling strategy, trajectory optimization

## Abstract

**Highlights:**

**What are the main findings?**

- A bi-level collaborative framework integrating PSO and Bi-RRT is proposed for AGV multi-target path planning in complex logistics environments.
- Adaptive sampling, local-density suppression, and lightweight online parameter adjustment strategies are introduced to improve planning efficiency and reduce redundant exploration.

**What are the implications of the main findings?**

- The proposed framework improves the balance between global planning efficiency and local path feasibility in cluttered environments.
- The method provides a practical solution for trajectory optimization and multi-target path planning of AGV in warehouse and logistics applications.

**Abstract:**

Multi-target path planning for Automated Guided Vehicle (AGV) in complex logistics environments requires balancing planning efficiency, obstacle avoidance capability, and trajectory smoothness. To address these challenges, this paper proposes a bi-level collaborative framework integrating Particle Swarm Optimization (PSO) with the Bidirectional Rapidly Exploring Random Tree (Bi-RRT). The framework unifies adaptive sampling, online parameter optimization, and trajectory smoothing within a single planning architecture. Specifically, the framework constructs a five-dimensional particle encoding that includes the expansion step size and multi-level strategy switching thresholds. During the Bi-RRT expansion process, an expansion-failure-driven adaptive sampling mechanism is introduced to enhance search performance in cluttered environments, while local-density-based suppression and directional dispersion are employed to reduce redundant exploration. In addition, a lightweight PSO-based monitoring mechanism enables online adaptive parameter adjustment. For multi-target scheduling, a greedy heuristic based on a hybrid weighted graph determines the visitation sequence. Trajectory smoothness is further improved using cubic B-spline interpolation combined with bounded perturbation optimization. Experimental results demonstrate that the proposed framework improves planning efficiency while maintaining stable performance across environments with different obstacle densities. These results demonstrate the effectiveness of the proposed framework for multi-target AGV path planning in complex warehouse environments.

## 1. Introduction

AGV is widely used in warehousing and logistics [1]. Path planning plays a key role in system efficiency and safety, aiming to generate smooth, collision-free trajectories from the start to the goal in complex environments. Similar requirements also arise in UAV swarms [2], Unmanned Surface Vehicles (USVs) [3], harvesting manipulators [4], surveying robots [5], and hull-maintenance robots [6], making path planning a fundamental component of autonomous navigation for various mobile platforms.

Early studies mainly relied on graph-search and meta-heuristic algorithms. In graph-based methods, variants of the A algorithm, such as Q-learning-based DP-A [7] and RLHA* integrated with neural networks [8], improve heuristic functions and enhance adaptability in unknown environments. The GPTA* algorithm introduces global guidance to improve search performance in grid-based maps [9]. Meta-heuristic algorithms have also been widely applied to handle multi-objective optimization and complex constraints. Genetic Algorithm (GA) is commonly used for task scheduling [10], while PSO is applied to path conflict resolution [11]. Recent advances in swarm intelligence, such as hybrid multi-swarm PSO [12] and large-scale multi-swarm scheduling strategies [13], further enhance global search capability and robustness in complex optimization problems. Continuous ant colony optimization (ACOPA R) helps avoid local optima through random-walk behavior [14], and multi-agent reinforcement learning combined with GA has been used to solve the Traveling Salesman Problem (TSP) [15]. However, these methods often show limited computational efficiency in high-dimensional spaces and densely cluttered environments.

Sampling-based Rapidly Exploring Random Tree (RRT) and its variants are widely used for path planning in high-dimensional and complex environments. However, these algorithms often exhibit slow convergence and unstable initial trajectories in narrow passages. Recent studies address these limitations by improving different components of the algorithm. At the sampling stage, Fast-RRT [16] and F-RRT [17] improve efficiency through refined parent-node selection, while SOF-RRT [18], FHQ-RRT* [19], Agile-RRT* [20], and GD-RRT* [21] reduce redundant exploration using spatial probability weighting, sparse sampling, or Gaussian-based methods. In addition, hybrid optimization strategies have been introduced to enhance AGV path planning performance, such as KGWO-based methods that integrate multiple optimization mechanisms [22].

During the search process, several methods adopt bidirectional expansion strategies. PSBi-RRT [23] introduces probability smoothing. APF-based methods guide the search direction using artificial potential fields [24], while goal-biased APF-RRT [25] further improves exploration efficiency. Hybrid-RRT [26] enhances the search process through hybrid sampling.

Kinematic constraints are considered in several methods. KB-RRT [27] incorporates motion constraints into tree expansion. Methods designed for Ackermann steering and Dubins vehicles, such as RRTASV [28] and 3PDP [29], address path curvature continuity to support feasible vehicle motion.

Recent studies have widely applied Reinforcement Learning (RL) to dynamic path planning. The expected mean gamma incremental RL [30] and the 2D LiDAR-based VFH-QL [31] incorporate state preference information to address multi-objective planning in environments without prior knowledge. Digital Twin (DT)-assisted federated RL [32] uses predictions from virtual environments to compensate for data synchronization delays in real-world systems. In addition, RL has been combined with geometric risk evaluation to address the scheduling of UAV edge computing nodes [33].

Existing methods, typically designed for constrained scenarios, often struggle to simultaneously balance search efficiency, trajectory smoothness, and operational safety for AGV navigating complex, dynamic industrial environments. Conventional algorithms are also prone to local optima in intricate terrains or generate paths with excessive inflection points, which degrade kinematic stability and increase energy consumption.

This paper proposes a bi-level multi-objective path planning framework to address these limitations. While recent studies have explored methods such as Grey Wolf Optimization with A*, Conflict-Based Search, and Artificial Potential Fields for multi-target path planning, these conventional approaches often combine existing algorithms or rely on empirically fixed heuristics. In contrast, the proposed method establishes a dynamic coupling between high-dimensional improved PSO and adaptive Bi-RRT. The bi-level architecture treats parameter tuning as an online process by introducing a continuous feedback mechanism rather than treating it as an isolated offline task.

At the upper level, PSO performs dynamic optimization over a five-dimensional hyperparameter set using dimensional decoupling and stagnation-aware updates. The resulting parameters continuously guide the lower-level Bi-RRT, which adopts a multi-level sampling strategy driven by expansion failures to improve exploration efficiency and escape local optima. Based on the generated geometric paths, trajectory smoothness is further enhanced using a range-jitter-constrained PSO combined with cubic B-splines, ensuring kinematic feasibility.

Finally, a mixed-weight graph model is employed to determine the visiting sequence for multi-target tasks. The proposed framework integrates path generation, smoothing, and task sequencing within a unified structure, demonstrating strong adaptability and improved performance compared with conventional hybrid planners. Table 1 summarizes the novelty of the proposed framework in contrast to the aforementioned recent methods.

## 2. Problem Formulation and Environment Modeling

### 2.1. Problem Statement and Assumptions

To analyze the efficiency and smoothness of the proposed bi-level collaborative path planning mechanism, the operational environment of a large-scale logistics center is simplified. The problem formulation is based on the following assumptions:

Single-AGV Setting: The study considers multi-target path planning for a single AGV. Multi-agent coordination and complex scheduling interactions are not included.

Static Environment: The warehouse is modeled as static. Dynamic elements commonly present in real systems—such as moving obstacles, other vehicles, and temporarily placed goods—are not considered.

Point-Mass Model: The AGV is represented as a point mass. Vehicle dynamics and kinematic constraints, including inertia, non-holonomic motion, and minimum turning radius, are not explicitly modeled. The focus is placed on geometric path-planning performance.

Unrestricted Free Space: The AGV can move freely within the free space $\Omega_{\mathrm{free}}$, provided that the corresponding grid cell is unoccupied.

The characteristics of the considered problem are summarized as follows. The inputs include the grid-based environment map S(x,y), the AGV starting position $v_{0}$, and the predefined set of target nodes V. The outputs consist of the target visiting sequence π and the corresponding collision-free B-spline trajectory C(u). The optimization objectives are to minimize the global multi-target traversal cost J(π), reduce local path search time, and improve trajectory smoothness while maintaining collision-free navigation.

### 2.2. Grid-Based Environment Representation

The warehouse environment is represented using a grid-based model. The workspace is defined as a two-dimensional domain Ω and discretized into an M × M uniform grid. The coordinate origin is located at the lower-left corner, and each grid cell has a unit side length.

Environmental information is encoded using Boolean values. A state function S(x,y)∈{0,1} is defined, where S(x,y)=1 denotes an occupied cell belonging to the obstacle region $\Omega_{\mathrm{obs}}$, and S(x,y)=0 indicates that the cell lies in the free space $\Omega_{\mathrm{free}}$.

**Figure 1:**
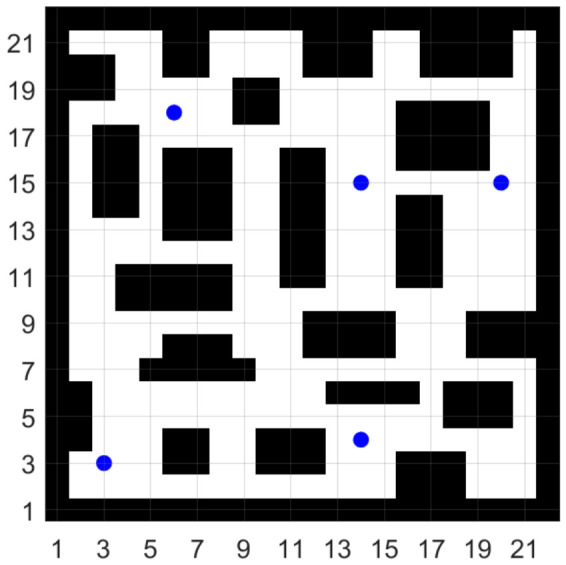
Grid-based representation of the warehouse environment. The blue markers indicate the target nodes, the white regions represent the free space, and the black regions represent the obstacles.

Since the AGV is modeled as a point mass, obstacle clearance is implicitly handled by the grid structure. No additional safety margin is incorporated into the distance cost function. Some grid cells are designated as task target points, shown as blue markers in Figure 1.

## 3. Improved PSO-Bi-RRT Bi-Level Collaborative Algorithm

Traditional adaptive RRT methods often rely on static goal biasing or distance-based step-size adjustment. The improved Bi-RRT in this study introduces a closed-loop feedback mechanism that integrates failure-driven state transitions, density-based dynamic repulsion, angular constraints, and a continuous expansion strategy. This mechanism enables the algorithm to adapt its search behavior to local geometric features rather than relying on purely random exploration.

### 3.1. Improved Bi-RRT Algorithm

(1) Multi-level sampling strategy driven by expansion failure count

Existing RRT methods often rely on fixed empirical probability distributions for sampling. This method introduces a multi-level state transition mechanism. A dynamic multi-level adaptive sampling strategy based on the expansion failure count $N_{fail}$ is proposed to improve the expansion efficiency of the random tree in complex environments. The reference direction $\theta_{ref}$ is defined from the node $X_{near}$, closest to the goal, toward $X_{goal}$.

The algorithm is divided into the following four adaptive stages:

(1) Goal-directed Stage ($N_{fail}\leq 2$):

In collision-free regions along the reference direction, a goal-directed greedy strategy accelerates tree expansion. The goal node $X_{goal}$ can be selected as the sampling point, with sampling also performed within a small neighborhood around $\theta_{ref}$, promoting rapid convergence toward the goal.

(2) Locally directed expansion Stage ($2<N_{fail}\leq N_{stage1}$):

When $N_{fail}$ exceeds the threshold of the previous stage, the presence of small obstacles ahead of the tree is inferred. Sampling is then restricted to a ±80° sector centered on the reference direction $\theta_{ref}$. This specific angular sector was geometrically selected to ensure a forward-biased expansion while strictly preventing inefficient backward or purely perpendicular tree growth. This sector is divided into k equal sub-sectors (k=4), within which candidate sampling points are generated with small Gaussian perturbations. After collision checking, the candidate with the minimum Euclidean distance to $X_{goal}$ is selected as the expansion target.

(3) Wide-area detour expansion Stage ($N_{stage1}<N_{fail}\leq N_{stage2}$):

When $N_{fail}$ exceeds the threshold of the previous stage, a wider obstacle ahead is inferred. The sampling range is expanded to a ±120° sector. These thresholds and subsequent allocation probabilities were determined through extensive preliminary simulations to optimally balance greedy exploitation and necessary random exploration. With a probability of 30%, samples are drawn within the ±80° region to maintain a baseline goal-directed progression, while with probability 70%, sampling is performed in the lateral regions spanning ±80° to ±120°, facilitating detours around obstacles.

(4) Global Escape expansion Stage ($N_{fail}>N_{stage2}$):

When all preceding expansion attempts fail, the current node is considered to be trapped in a deep local minimum, such as a U-shaped configuration. Sampling is extended to the full 360° space. The sampling distribution is biased toward the rear region to promote backtracking and escape from spatial traps; a probability of 10% is assigned to the forward ±80° region, 20% to the regions between ±80° and ±120°, and the remaining 70% to the regions between ±120° and ±180°.

Preliminary experiments identified $N_{stage1}\in [3,8]$ and $N_{stage2}\in [3,8]$ as suitable optimization ranges. Values below 3 tend to trigger excessive strategy switching, whereas values above 15 are frequently associated with prolonged search stagnation in highly constrained regions, such as U-shaped obstacles. The upper-level PSO performs online optimization within these ranges, reducing the reliance on manually selected threshold values.

**Figure 2:**
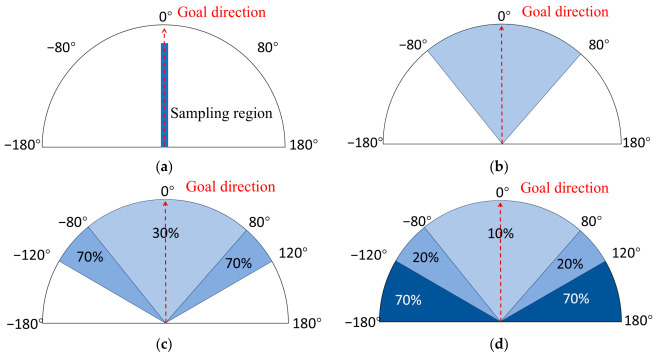
Schematic of the four-level adaptive sampling sector based on the number of expansion failures. (**a**) Goal-directed Stage. (**b**) Locally directed expansion Stage. (**c**) Wide-area detour expansion Stage. (**d**) Global Escape expansion Stage. The regions shaded in varying shades of blue represent the feasible sampling range for the nodes.

Figure 2 illustrates the multi-level adaptive sampling strategy guided by the number of expansion failures:

(2) Dynamic Invalid-Radius Suppression Mechanism Based on Local Density

A newly generated candidate node $X_{new}$ is accepted if its distance to any existing tree node $X_{i}$ satisfies the condition $min∥X_{new}\;-X_{i}\;∥\geq R_{dyn}$. In this context, the adaptive invalid radius $R_{dyn}$ is defined as:

(1) $$R_{dyn}(X_{new})=R_{base}\cdot (1+\eta \cdot \frac{N_{local}}{N_{total}})$$

(2) $$R_{base}=S\;\cdot \;\kappa$$

where $R_{base}$ denotes the base invalid radius, S represents the expansion step size, and κ∈[0.2,0.7] is the invalid-radius coefficient. Additionally, $N_{total}$ indicates the total number of nodes in the tree, $N_{local}$ corresponds to the number of nodes within a local neighborhood centered at $X_{new}$ with radius $R_{base}$, and η∈[3,8] is the density penalty factor.

In sparse regions, $R_{dyn}\approx R_{base}$, allowing the algorithm to perform fine-grained exploration. In contrast, in high-density regions, $R_{dyn}$ increases, and candidate nodes falling within the invalid radius are excluded from connection.

(3) Reference-Angle Dispersion Optimization

Existing Bi-RRT algorithms typically rely solely on Euclidean distance to maintain node diversity and rarely consider the angular distribution of branches, which tends to produce redundant parallel paths. To address the issue, this study introduces an angle-repulsion constraint to enhance the diversity of search directions. The algorithm maintains a set of directions, $\Phi =\{\phi_{1},\phi_{2},...,\phi_{n},\}$, corresponding to existing branches within the neighborhood of the nearest node $X_{near}$. For each newly generated candidate sampling angle, the minimum angular distance to any direction in the set Φ is computed as:

(3) $$\Delta \theta_{min}=min|\theta_{rand}\;-\phi_{j}\;|\;\;,(\phi_{j}\in \Phi )$$

where $\Delta \theta_{min}$ denotes the current minimum angular, $\delta_{th}=25{}^{\circ}$ is the angle-repulsion threshold, and $\theta_{rand}$ represents the candidate sampling angle. If $\Delta \theta_{min}<\delta_{th}$, the direction is considered overly congested, and $\theta_{rand}$ is forcibly reset.

(4) Continuous Expansion Based on Hysteresis Decision and Directional Inertia

Conventional Bi-RRT relies on single-step expansion, which is prone to path oscillations in narrow passages. This strategy introduces a continuous expansion mechanism to accelerate tree growth and improve obstacle avoidance stability.

(1) To utilize directional inertia for accelerating the growth of the random tree, when the algorithm is in the obstacle-avoidance stage ($N_{fail}\geq N_{stage1}$) and a new node $X_{new}$ is successfully generated, a greedy probing expansion is performed five times along the current sampling direction. If any candidate node encounters a collision or exceeds the boundary, the inertia-based expansion is immediately terminated.

(2) Single-step sampling may be insufficient for escaping obstacle regions. A hysteresis decision mechanism is introduced to maintain the current sampling strategy until a stable exit is achieved. After the inertia-based expansion, a multi-step straight-line collision check is performed from the new node toward the global goal $X_{goal}$. If the path is collision-free, the branch of the random tree is considered to have exited the obstacle, $N_{fail}$ is reset to zero, and the algorithm returns to the direct-steering mode. Otherwise, $N_{fail}$ remains unchanged, and the lateral detour mode is maintained in the next iteration.

### 3.2. Multi-Strategy Improved PSO Algorithm

**Figure 3:**
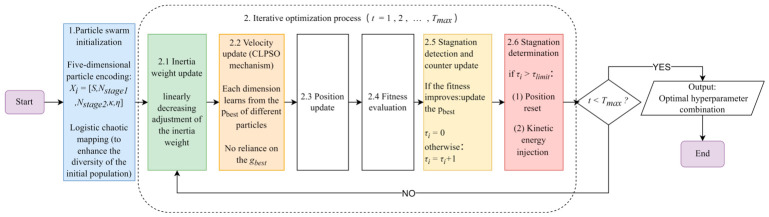
Flowchart of the Multi-Strategy Improved PSO Algorithm.

Due to the high coupling between parameters such as the expansion step size and the sampling-angle constraint threshold, the presence of multiple local optima in the solution space, and the strong stochastic noise inherent in the evaluation process, conventional PSO is prone to premature convergence in the context of Bi-RRT algorithms. To address these limitations, this study proposes an improved PSO algorithm that integrates high-dimensional particle encoding, logistic mapping, a comprehensive CLS mechanism, and a stagnation-based reset strategy. Figure 3 (Flowchart of the Multi-Strategy Improved PSO Algorithm) shows a detailed flowchart of the proposed multi-strategy improved PSO algorithm. Colored highlights indicate the components that contribute to the improvement of the search process. Furthermore, a linearly decreasing scheme is employed to dynamically adjust the inertia weight throughout the optimization process.

(4) $$\omega (t)=\omega_{max}-\frac{t}{T_{max}}(\omega_{max}-\omega_{min})$$

where $T_{max}$ denotes the maximum number of iterations, $\omega_{max}=0.9$, $\omega_{min}=0.4$, and t is the current iteration number.

(1) High-Dimensional Particle Encoding Structure

The position vector of the i-th particle, $X_{i}$, is defined as a five-dimensional hyperparameter set encompassing three categories of parameters: motion characteristics, decision thresholds, and spatial constraints.

(5) $$X_{i}=[S,N_{stage1},N_{stage2},\kappa ,\eta ]$$

where S∈[0.5,1.5] denotes the base expansion step size. The state transition thresholds $N_{stage1}$ and $N_{stage2}$ correspond to the conditions governing the transitions within the multi-level sampling strategy. The dynamic suppression parameters κ and η regulate the effective range of the density-based adaptive invalid radius $R_{dyn}$, where κ represents the base invalid-radius coefficient, and η denotes the density-penalty strength. The complete list of optimized parameters is summarized in Table 2.

(2) Particle Population Diversity Enhancement

The logistic chaotic map is employed to enhance the diversity of the particle population within the five-dimensional hyperparameter search space. The velocity update formula utilizes a CLS. CLPSO enables each particle, when updating its velocity across different dimensions, to learn from the individual historical bests of distinct particles in the population, without relying on global best guidance. This dimensional decoupling mechanism helps prevent the swarm from premature convergence to local optima.

(3) Stagnation-Detection-Based Population Diversity Maintenance

Each particle is equipped with a stagnation counter, $\tau_{i}$. During each iteration, when updating $p_{best}$, if the fitness value of particle i changes, the counter $\tau_{i}$ is reset to zero; otherwise, it is incremented by one. Once $\tau_{i}$ exceeds the predefined threshold $\tau_{limit}$, the particle is considered to have become trapped in a local optimum or to have lost its search capability. The following remedial actions are then applied to the particle to restore population entropy and guide the algorithm out of local stagnation:

1. Position Reset: The particle’s position is randomly regenerated within the solution space.

2. Kinetic Energy Injection: The particle is assigned a relatively large random initial velocity.

### 3.3. Full-Process Collaborative Mechanism of Improved PSO and Bi-RRT

**Figure 4:**
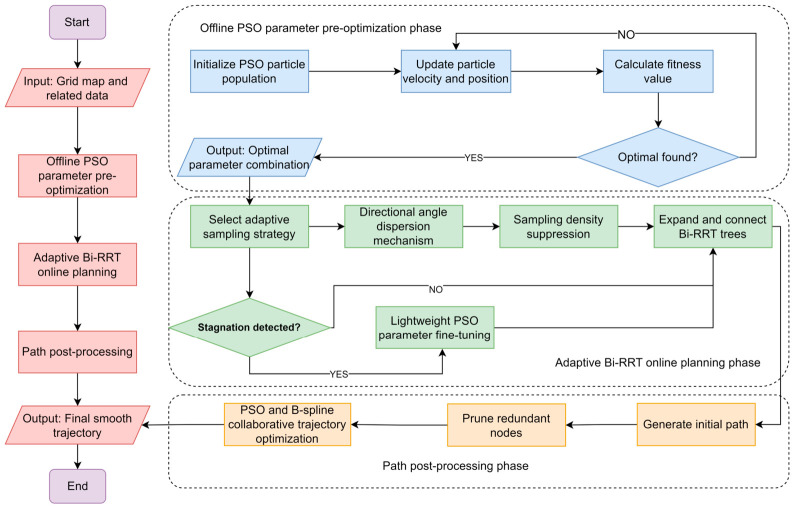
Flowchart of the full-process collaborative mechanism of PSO and Bi-RRT.

While Bi-RRT provides efficient exploration in high-dimensional spaces, its performance is sensitive to parameter settings and may suffer from local search stagnation in complex warehouse environments. PSO, in contrast, offers strong global search capability and adaptive optimization characteristics. The proposed two-layer collaborative framework integrates PSO with Bi-RRT, enabling dynamic parameter adjustment during planning and alleviating search stagnation in complex environments. The overall workflow of the framework is illustrated in Figure 4.

#### 3.3.1. Definition of the Fitness Function

To mitigate the influence of stochasticity in the Bi-RRT algorithm on parameter optimization, a mean-based evaluation strategy is adopted during the fitness assessment stage. For each parameter combination $X_{i}$ represented by a particle, K independent Bi-RRT path planning trials are executed consecutively, and the average path cost, average computation time, and success rate are computed. The fitness function is then formulated as follows:

(6) $$Fitness(X_{i})\;=\;\alpha \;\cdot \;{\bar{T}}_{search}\;+\;\beta \;\cdot \;{\bar{L}}_{path}\;+\;\gamma \;\cdot {\;\bar{N}}_{collision}\;+\;\;P_{failure}$$

where α, β, and γ are weighting coefficients assigned values of 0.4, 0.4, and 0.2, respectively. ${\bar{T}}_{search}$, ${\bar{L}}_{path}$, and ${\bar{N}}_{collision}$ denote the average path planning time, path length, and number of collisions over K Bi-RRT trials, respectively, while $P_{failure}$ represents a penalty term.

To balance evaluation accuracy and computational efficiency, two evaluation modes are defined:

1. Offline Evaluation Mode: A mean-based strategy with K=3 trials is employed.

2. Online Evaluation Mode: A fast evaluation strategy with K=1 is adopted.

#### 3.3.2. Offline and Online Lightweight Improved PSO Mechanism

(1) Offline Pre-optimization of Environment-Adaptive Parameters

Prior to the execution of the improved Bi-RRT algorithm, the PSO algorithm is first initialized to perform offline optimization of the parameter set $\left[S,N_{stage1},N_{stage2},\kappa ,\eta \right]$, based on the obstacle-density characteristics of the current warehouse map. This process aims to identify an optimal combination of the expansion step size, strategy-switching thresholds, and density suppression coefficients in advance.

(2) Online Hybrid Trigger Mechanism and Lightweight Fine-tuning

During the Bi-RRT expansion process, a hybrid monitoring mechanism is designed to continuously assess the search state and detect potential deadlocks. When the following condition is satisfied, the PSO-based optimization of Bi-RRT is triggered:

(1) Excessive Collision Rate: If the proportion of collision failures $\rho_{coll}$ exceeds a predefined threshold within 25 consecutive expansion attempts, the PSO mechanism is activated.

(7) $$\rho_{collision}=\frac{N_{collision}}{25}\;\cdot 100\%>70\%$$

where $\rho_{collision}$ denotes the collision rate and $N_{collision}$ represents the number of collision events. Based on extensive simulation-based calibration, the collision-rate threshold is set to 70%.

(2) Expansion Stagnation: The growth status of the search trees is continuously monitored. If, over 50 consecutive iterations, the node growth rates, $p_{grow}$, of both trees fall below a predefined threshold, the algorithm is considered to have entered a stagnation state:

(8) $$p_{grow}=\frac{N_{total}(t)\;-\;N_{total}(t-50)}{N_{total}(t-50)}\cdot 100\%<5\%$$

where $p_{grow}$ denotes the node growth rate and $N_{total}(t)$ represents the total number of nodes in the tree at iteration t. Based on extensive simulation-based calibration, the node growth rate threshold is set to 5%.

(3) Time-Limit Constraint Early Warning: If the current iteration exceeds 60% of the maximum allowed iterations and the two trees have not yet connected, the algorithm is considered to be potentially trapped in a local optimum.

### 3.4. Path Post-Processing and Trajectory Smoothness Optimization

This study proposes a PSO-based trajectory collaborative optimization strategy with range-jitter constraints. Redundant nodes in the initial path are first removed using a line-of-sight method, and the resulting path nodes are mapped to the initial control points of a B-spline curve. Subsequently, the PSO algorithm performs fine-tuning of the control points within their respective searchable regions to identify the optimal set of B-spline control points.

1. Jitter Space Construction and Particle Encoding

Let the set of path nodes after redundancy elimination be denoted as $P=\{p_{1},...,p_{n}\}$. Except for the start and end nodes, which are fixed, the searchable region of each intermediate node $p_{i}$ is defined as a circular domain $\Omega_{i}$ centered at its original position:

(9) $$\Omega_{i}=\{(x,y)\in R^{2}|\|(x,y)-p_{i}\|\leq R_{jitter}\}$$

where $R_{jitter}$ denotes the radius of the searchable domain for each node. $R_{jitter}$ is set to 0.3S, where S is the Bi-RRT expansion step size. This bounded-jitter constraint limits excessive deviation from the original feasible path while preserving sufficient optimization flexibility.

**Figure 5:**
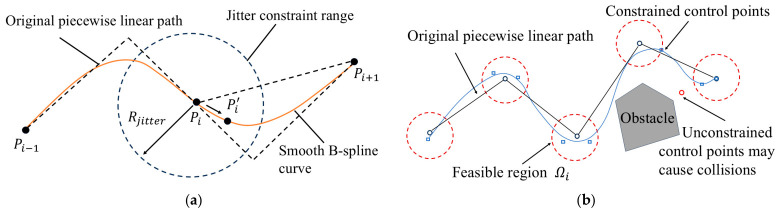
Collaborative B-spline trajectory optimization process based on range jitter constraints. (**a**) Local geometric definition. (**b**) Global path optimization scenario.

If a control point enters an obstacle region during particle updates, the corresponding particle is either projected onto the boundary of the feasible domain or reset to its last valid position. Furthermore, after each control-point update, the resulting B-spline trajectory is uniformly discretized and evaluated for collisions. Trajectories that intersect obstacles are assigned a large penalty during fitness evaluation, preventing them from being selected as feasible solutions. Consequently, the optimization process is restricted to collision-free trajectories, ensuring obstacle avoidance throughout trajectory generation. This optimization process is illustrated in Figure 5.

2. Collaborative Fitness Evaluation

The fitness function $F_{smooth}$ directly evaluates the B-spline trajectory C(u) induced by the current control points, thereby jointly optimizing control point placement and trajectory quality. The evaluation integrates path length, smoothness, and safety:

(10) $$F_{smooth}=w_{len}\cdot L\left(C\right)+w_{smooth}\cdot \sum_{k}(1-v_{k}\cdot v_{k+1}{)}^{2}+P_{obs}\left(C\right)$$

where L(C) denotes the arc length of the generated curve, and $P_{obs}(C)$ represents a collision penalty term defined via a penalty function. This term is assigned a sufficiently large value, for example, $10^{10}$, when any sampling point collides with an obstacle. $v_{k}$ denotes the unit tangent vector at sampling point k, where k indexes the discretized curve. The weights $w_{len}$ and $w_{smooth}$ correspond to the curve length and smoothness terms, respectively, with $w_{smooth}\gg w_{len}$; in this study, they are set to $w_{smooth}=50$ and $w_{len}=1$.

### 3.5. Multi-Target Visit Sequence Planning Based on Hybrid Graph Weights

A complete graph model with hybrid edge weights is constructed, and a greedy algorithm is employed to determine an AGV operation sequence that minimizes both energy consumption and time cost.

(1) Problem Modeling and Hybrid Weight Strategy

The AGV task scheduling problem is formulated as a complete undirected weighted graph G=(V,E). The vertex set $V=\{v_{0},v_{1},\ldots ,v_{N}\}$ consists of the starting node $v_{0}$ and N-1 target locations to be visited.

A two-level weighting scheme is adopted for the edge weights $W_{ij}$. If the straight-line path between nodes $v_{i}$ and $v_{j}$ is collision-free, $W_{ij}$ is defined as the Euclidean distance between them. Otherwise, an obstacle-avoiding path is generated using the improved PSO and Bi-RRT algorithm, and the corresponding path length is assigned to $W_{ij}$. If path planning fails, $W_{ij}$ is set to infinity.

(2) Construction of the Comprehensive Obstacle-Avoidance Cost Matrix

An environment complexity penalty factor is introduced to adjust the physical path length, thereby constructing a composite cost matrix C that reflects traversal cost. For non-line-of-sight paths, the matrix elements are defined as follows:

(11) $$C_{ij}=L_{ij}+c_{base}\cdot (\alpha +\beta )$$

(12) $$c_{base}=L_{ij}-d_{ij}$$

(13) $$d_{ij}={∥v_{i}-v_{j}∥}_{2}$$

(14) α=1+ρ

(15) $$\rho =N_{obs}/N_{total}$$

(16) $$\beta =1+0.1\cdot N_{point}$$

where $C_{ij}$ denotes the element of the composite cost matrix, and $L_{ij}$ represents the length of the collision-free obstacle-avoiding path between nodes $v_{i}$ and $v_{j}$. The $c_{base}$ denotes the baseline detour cost incurred by obstacle avoidance. The Euclidean distance between the two nodes is denoted by $d_{ij}$. The coefficient α is the obstacle density factor, where $N_{total}$ denotes the total number of grid cells along the straight-line path between two nodes, and $N_{obs}$ represents the number of obstacle-occupied grid cells among them. The parameter β is the path complexity coefficient, and $N_{point}$ denotes the number of turning points along the path where the turning angle satisfies Δθ>30°.

(3) Greedy-Based Visit Sequence Optimization

Based on the cost matrix C, the multi-target planning problem is formulated as an optimization over permutations $\pi =(\pi_{0},\pi_{1},\ldots ,\pi_{N})$, with the objective of minimizing the global operational cost function J(π):

(17) $$\min J(\pi )=\sum_{k=0}^{N-1}C_{\pi_{k},\pi_{k+1}}$$

The solution is subject to the constraint $\pi_{0}=v_{0}$, and $\left\{\pi_{1},\ldots ,\pi_{N}\right\}$, which forms a permutation of the target set. Considering that the preceding Bi-RRT stage has already consumed part of the computational resources and that real-time performance is critical in warehouse scheduling, a nearest-neighbor greedy strategy is adopted to obtain a near-optimal sequence. The time complexity of this strategy is $O(N^{2})$, which enables millisecond-level responsiveness for multi-target tasks.

## 4. Experiment

### 4.1. Simulation Platform and Parameter Settings

Simulation experiments are conducted in MATLAB R2021a to evaluate the effectiveness and robustness of the proposed algorithm in complex warehouse environments. All experiments are performed on a laptop equipped with an Intel Core i7-13700H processor and 16 GB of memory. The proposed framework is compared with three conventional algorithms (Bi-RRT, RRT, and RRT-Connect), a Bi-RRT variant with offline PSO, and an improved Bi-RRT without PSO. In addition, two representative heuristic-guided methods, namely Artificial Potential Field-guided Bi-RRT (APF-Bi-RRT) and Grey Wolf Optimizer-guided Bi-RRT (GWO-Bi-RRT), are included to evaluate the effectiveness of different hybrid guidance mechanisms. All evaluated algorithms are run under the same computational budget and identical hardware configurations to ensure a fair and reproducible comparison.

A unified set of fundamental parameters is applied across all methods. The expansion step size is set within the range [0.5, 1.5], and the connection radius is defined to be equal to the step size. The maximum number of iterations is set to 4000, 5000, 10,000, 15,000, and 15,000 for the five test scenarios, respectively.

A consistent stopping criterion is applied: each algorithm terminates upon finding a valid collision-free path between the start and goal or upon reaching the maximum number of iterations, in which case the run is recorded as a failure.

Simulations are conducted on three maps with different obstacle scales, along with two constrained scenarios, namely a U-shaped obstacle and a narrow passage. Performance is evaluated in terms of path length, sampled node count, and computation time. A statistical analysis is conducted to evaluate performance improvements and account for the stochastic nature of sampling-based planners. All algorithms are independently executed 50 times under identical configurations. Results are reported as mean ± standard deviation (SD) to reflect performance variability and stability.

Given that RRT-based results may exhibit non-normal distributions and heteroscedasticity, a non-parametric Wilcoxon rank-sum test is adopted instead of a parametric test. This test assesses the significance of performance differences between the proposed method and baseline algorithms. For all comparisons, the results yield *p* < 0.01, indicating statistically significant performance differences.

### 4.2. Comparison of Path Planning Trajectories Among Different Algorithms in Complex Warehousing Environments

**Figure 6:**
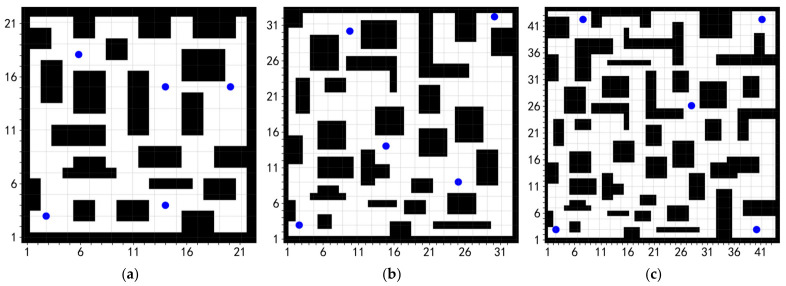
Environment A: Grid map schematic. (**a**) 22 × 22 Grid Map. (**b**) 33 × 33 Grid Map. (**c**) 44 × 44 Grid Map. The blue markers indicate the target nodes, the white regions represent the free space, and the black regions represent the obstacles.

Environment A includes three obstacle scenarios: sparse, moderate, and dense. Five target nodes are specified, resulting in a total of ten undirected test paths. For visual clarity, only the planning process for the node pair with the maximum Euclidean distance is illustrated, while evaluation is performed using data from all ten paths. For completeness, detailed trajectory visualizations for all independent planning instances in the representative 44 × 44 dense obstacle scenario are provided in the Supplementary Materials (Figures S1–S9 and Table S1). The expansion of the start and goal trees is represented by green and blue lines, respectively. The three environments are shown in Figure 6, where blue markers indicate the target nodes.

**Figure 7:**
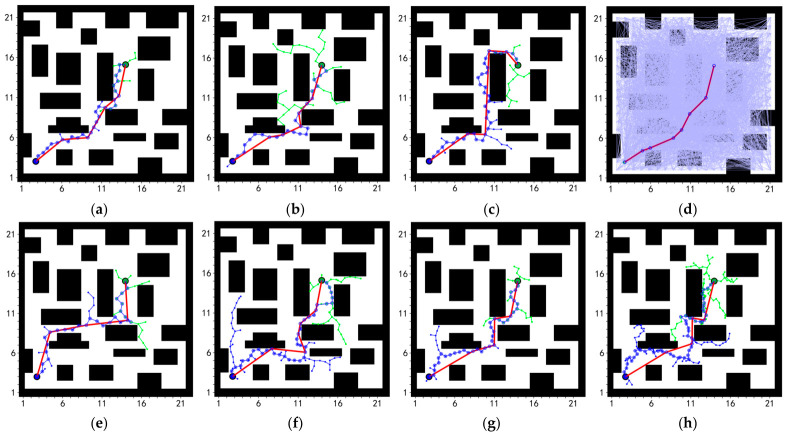
Scenario A: 22 × 22 sparse obstacle scenario. (**a**) Proposed algorithm. (**b**) Single offline PSO-optimized Bi-RRT. (**c**) Improved Bi-RRT without PSO. (**d**) RRT* algorithm. (**e**) RRT-Connect algorithm. (**f**) Bi-RRT algorithm. (**g**) APF-Bi-RRT algorithm. (**h**) GWO-Bi-RRT algorithm. The blue color indicates the start tree, the green color represents the goal tree, and the red color represents the optimized path trajectory.

**Figure 8:**
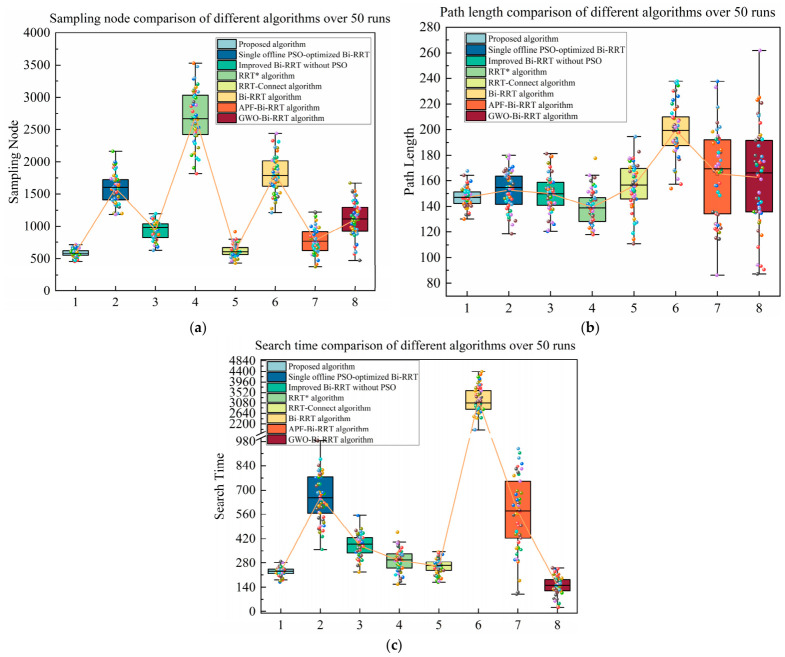
Scenario A: Comparison curves of 22 × 22 simulation data. (**a**) Sampling node comparison of different algorithms over 50 runs. (**b**) Path-length comparison of different algorithms over 50 runs. (**c**) Search time comparison of different algorithms over 50 runs.

**Figure 9:**
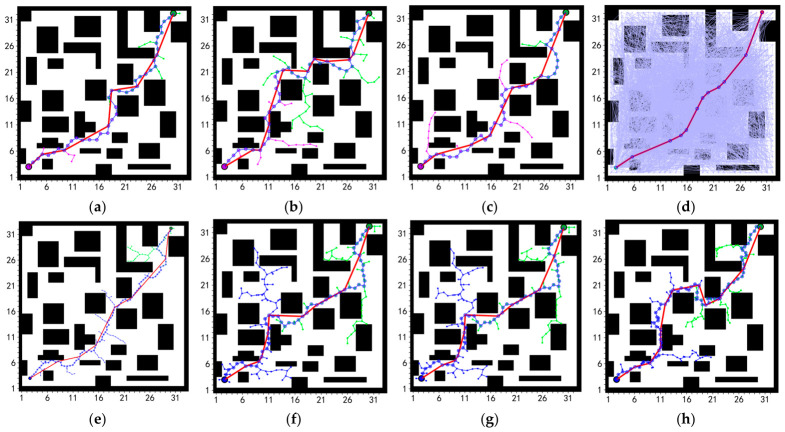
Scenario A: 33 × 33 medium obstacle scenario. (**a**) Proposed algorithm. (**b**) Single offline PSO-optimized Bi-RRT. (**c**) Improved Bi-RRT without PSO. (**d**) RRT* algorithm. (**e**) RRT-Connect algorithm. (**f**) Bi-RRT algorithm. (**g**) APF-Bi-RRT algorithm. (**h**) GWO-Bi-RRT algorithm. The blue color indicates the start tree, the green color represents the goal tree, and the red color represents the optimized path trajectory.

**Figure 10:**
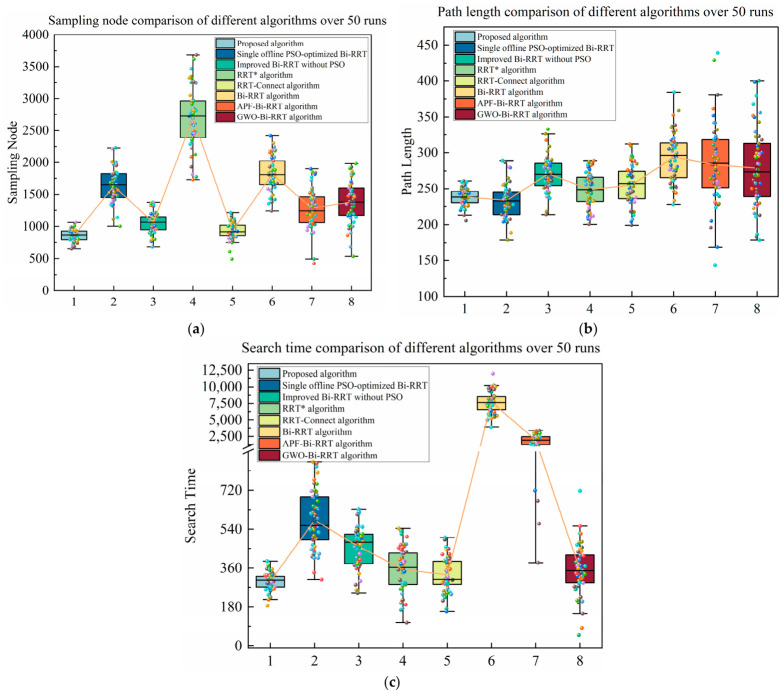
Scenario A: Comparison curves of 33 × 33 simulation data. (**a**) Sampling node comparison of different algorithms over 50 runs. (**b**) Path-length comparison of different algorithms over 50 runs. (**c**) Search time comparison of different algorithms over 50 runs.

**Figure 11:**
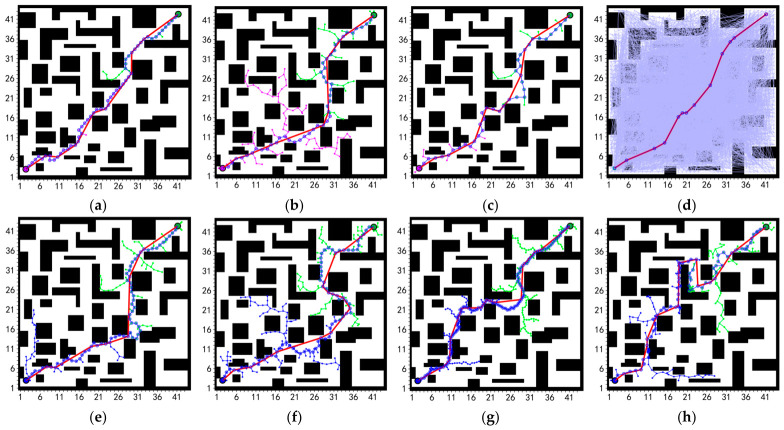
Scenario A: 44 × 44 dense obstacle scenario. (**a**) Proposed algorithm. (**b**) Single offline PSO-optimized Bi-RRT. (**c**) Improved Bi-RRT without PSO. (**d**) RRT* algorithm. (**e**) RRT-Connect algorithm. (**f**) Bi-RRT algorithm. (**g**) APF-Bi-RRT algorithm. (**h**) GWO-Bi-RRT algorithm. The blue color indicates the start tree, the green color represents the goal tree, and the red color represents the optimized path trajectory.

**Figure 12:**
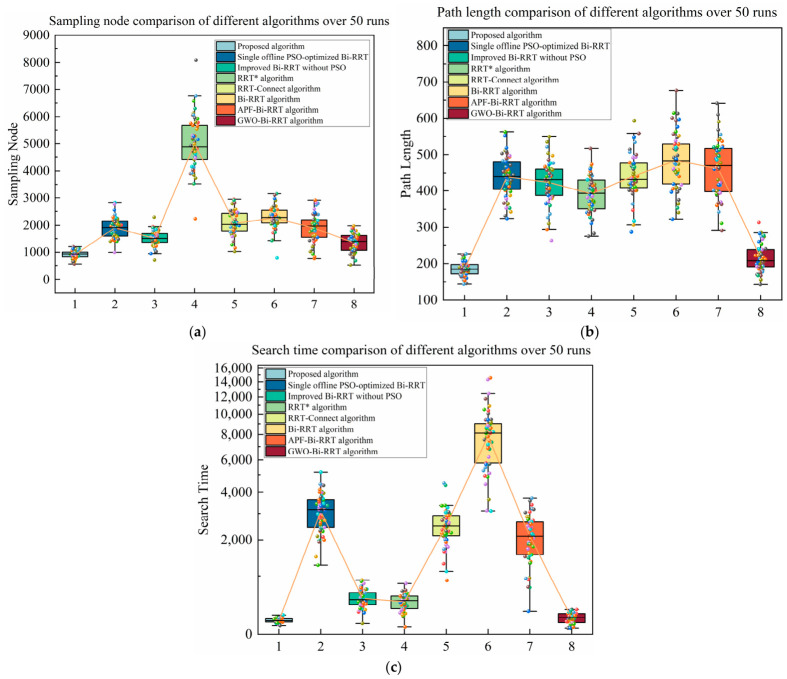
Scenario A: Comparison curves of 44 × 44 simulation data. (**a**) Sampling node comparison of different algorithms over 50 runs. (**b**) Path-length comparison of different algorithms over 50 runs. (**c**) Search time comparison of different algorithms over 50 runs.

The proposed algorithm combines dynamic sampling strategies with online PSO refinement, enabling the random tree to escape local minima and reducing ineffective sampling. The simulation trajectories of the various compared algorithms in the first scenario (the 22 × 22 sparse obstacle environment) are shown in Figure 7, and the corresponding parameter statistics curves are summarized in Figure 8. Furthermore, the simulation maps and statistical data for the second scenario (the 33 × 33 medium obstacle environment) are presented in Figure 9 and Figure 10. Finally, the path planning trajectories and data statistics for the third scenario (the 44 × 44 dense obstacle environment) are illustrated in Figure 11 and Figure 12. Experimental results show that the proposed method outperforms the benchmark algorithms in terms of average path length, sampled node count, and computation time. As environmental complexity increases, the proposed algorithm achieves more significant improvements in computational efficiency and obstacle avoidance.

The aggregated results over all planning instances and their corresponding instance-level evaluations are summarized sequentially according to the environmental complexity: Table 3 and Table 4 for the 22 × 22 scenario, Table 5 and Table 6 for the 33 × 33 scenario, and Table 7 and Table 8 for the 44 × 44 scenario. The instance-based analyses exhibit trends consistent with the aggregated statistics, indicating that the advantages of the proposed framework are not confined to a limited number of favorable cases. Despite performance variations caused by different obstacle configurations and planning tasks, the proposed method consistently demonstrates superior planning efficiency and reduced sampling requirements, thereby confirming its robustness and effectiveness across diverse environments.

**Figure 13:**
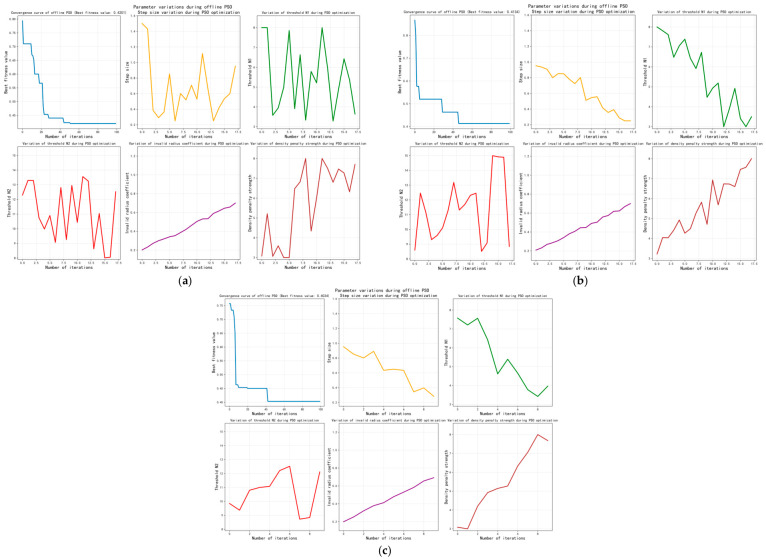
Scenario A: Optimization curves of various parameters in a 44 × 44 dense obstacle scenario. (**a**) Offline stage parameter optimization line chart. (**b**) Online starting point tree parameter optimization line chart. (**c**) Online endpoint tree parameter optimization line chart.

The introduced Logistic chaotic mapping strategy enhances population diversity, leading to faster convergence of the fitness curve (Figure 13a). Meanwhile, the PSO mechanism iteratively adjusts parameters to obtain an effective configuration for the current environment. When tree expansion stagnates in complex obstacle regions, an online monitoring mechanism activates a PSO-based adjustment to guide the search. This helps the algorithm escape local minima and traverse constrained regions (Figure 13b,c).

**Figure 14:**
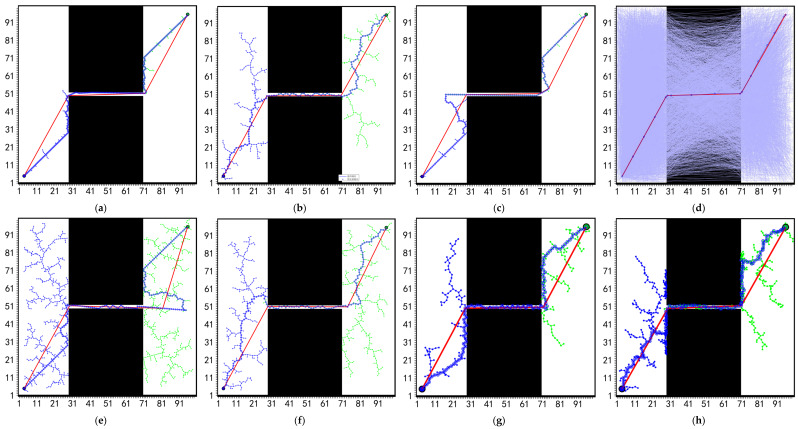
Scenario B: Narrow passage scenario. (**a**) Proposed algorithm. (**b**) Single offline PSO-optimized Bi-RRT. (**c**) Improved Bi-RRT without PSO. (**d**) RRT* algorithm. (**e**) RRT-Connect algorithm. (**f**) Bi-RRT algorithm. (**g**) APF-Bi-RRT algorithm. (**h**) GWO-Bi-RRT algorithm. The blue color indicates the start tree, the green color represents the goal tree, and the red color represents the optimized path trajectory.

**Figure 15:**
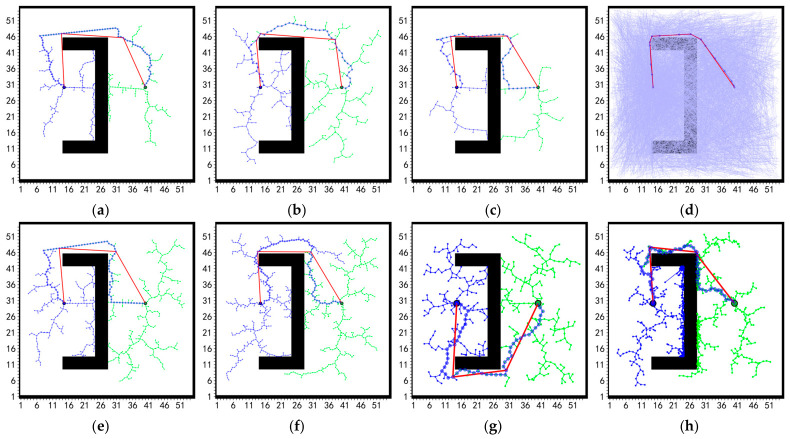
U-shaped obstacle scenario. (**a**) Proposed algorithm. (**b**) Single offline PSO-optimized Bi-RRT. (**c**) Improved Bi-RRT without PSO. (**d**) RRT* algorithm. (**e**) RRT-Connect algorithm. (**f**) Bi-RRT algorithm. (**g**) APF-Bi-RRT algorithm. (**h**) GWO-Bi-RRT algorithm. The blue color indicates the start tree, the green color represents the goal tree, and the red color represents the optimized path trajectory.

In extreme environments, the proposed algorithm produces fewer redundant expansions than the baseline methods (Figure 14 and Figure 15). The quantitative simulation results for the narrow passage scenario (Scenario B) are summarized in Table 9. In the U-shaped obstacle scenario (Scenario C), although RRT* occasionally yields shorter paths, as shown in Table 10, this is achieved at the cost of a higher number of sampled nodes. Overall results indicate that the proposed method outperforms the benchmark algorithms, particularly in reducing the average computation time and sampled node count.

### 4.3. Path Post-Processing and Multi-Target Visit Sequence Validation

**Figure 16:**
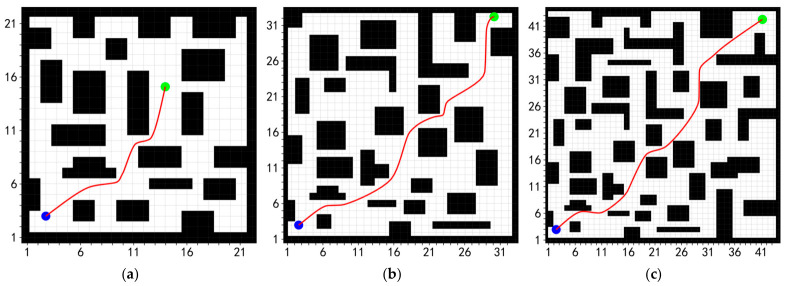
Scenario A: Post-processed paths of the proposed algorithm. (**a**) 22 × 22 sparse obstacle scenario. (**b**) 33 × 33 medium obstacle scenario. (**c**) 44 × 44 dense obstacle scenario. The blue and green points represent the two target points, and the red line represents the optimized path trajectory.

The effectiveness of the trajectory-smoothing strategy is evaluated using paths generated in the three configurations of Environment A (Figure 16). Under PSO-based bounded-jitter constraints, the cooperative B-spline optimization removes abrupt changes in piecewise-linear segments, producing a continuous and smooth curve. Compared with the original trajectory and the pruned path, the optimized trajectory exhibits improved smoothness.

**Figure 17:**
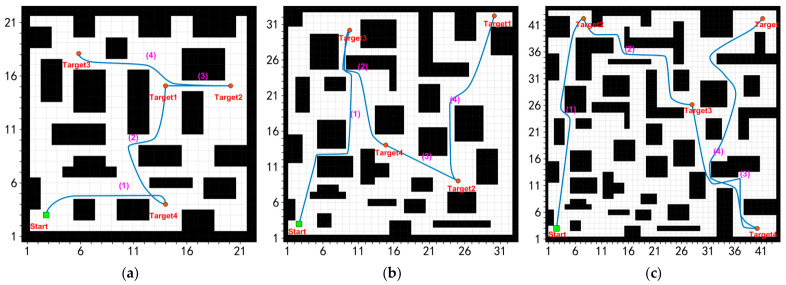
Scenario A: Optimal multi-target visit sequences. (**a**) 22 × 22 sparse obstacle scenario. (**b**) 33 × 33 medium obstacle scenario. (**c**) 44 × 44 dense obstacle scenario. The numbers indicate the sequence of visiting the multiple target points.

As shown in Figure 17, the optimal visiting sequence of the AGV in Environment A is given as follows:

Start“→” Target4“→” Target1“→” Target2“→” Target3;

Start“→” Target3“→” Target4“→” Target2“→” Target1;

Start“→” Target2“→” Target3“→” Target4“→” Target1.

## 5. Conclusions

This paper proposes a multi-objective path planning algorithm that integrates an improved PSO with an adaptive Bi-RRT to address the limitations of planning efficiency and obstacle avoidance in complex, high-density warehouse environments. Simulation results under different levels of complexity lead to the following conclusions:

1. This paper proposes a five-dimensional particle encoding scheme for kinematic features and decision thresholds and integrates PSO with Logistic chaotic mapping and CLS to achieve adaptive parameter tuning, improving the adaptability of the path planning algorithm across different environments.

2. This paper proposes an expansion strategy that integrates multi-level adaptive sampling, local density constraints, and orientation dispersion to adjust sampling directions and node distributions. Compared with conventional random expansion, the proposed method improves tree growth and alleviates stagnation during expansion. In addition, an online detection mechanism combined with a lightweight PSO supports real-time parameter tuning, improving the adaptability of the path planning framework in complex environments. Furthermore, statistical analysis indicates that the combination of the online detection mechanism and the lightweight PSO reduces search time and the number of sampled nodes. The proposed method exhibits lower SD and achieves statistical significance compared with all baseline algorithms, suggesting improved efficiency and stability.

3. This approach generates smooth, collision-free trajectories by combining a range-jitter-constrained PSO with cubic B-splines and obtains a cost-optimal visiting sequence using a greedy strategy on a mixed-weight graph.

Despite these results, the proposed method exhibits several limitations. First, although lightweight PSO enables online parameter tuning, the iterative evaluation of particle fitness introduces additional computational overhead, which may affect performance in large-scale grid maps. Second, the current framework assumes a static global environment and relies on prior map information. Future work should consider the application of AGVs in dynamic obstacle scenarios that are frequently encountered in real-world operations. Specifically, introducing dynamic RRT variants could significantly improve real-time obstacle avoidance capabilities. Further research will explore coordinated collision avoidance in multi-AGV systems by incorporating temporal conflict detection mechanisms to enhance autonomous operation in complex warehouse environments. In addition, greater attention should be given to employing multi-objective optimization to balance path length, computational efficiency, and safety when improving local path planning during collision avoidance.

## Figures and Tables

**Table 1:** Novelty comparison with recent studies.

Method	Search Paradigm	Parameter Adaptation	Local Minima Avoidance	Trajectory Feasibility
Dijkstra’s variants	Uninformed (blind) deterministic graph search	None	Strong completeness but suffers from extreme computational burden	Restricted to grid edges; kinematically unfeasible without smoothing
A* variants	Heuristic-guided discrete graph search	Fixed heuristic weights; lacks environmental adaptability	Performance and search speed severely degrade in cluttered traps	Generates paths with sharp turns; requires heavy post-processing
Conflict-Based Search	Conflict-based discrete search	None	Highly effective for multi-agent conflicts, less focused on spatial traps	Yields discrete paths; requires secondary geometric smoothing
APF-based methods	Artificial potential field continuous guidance	Limited to static field coefficients	Highly prone to local oscillations and deadlocks (e.g., U-shaped obstacles)	Implicitly smooth but lacks strict clearance guarantees
GWO-based methods	Metaheuristic-guided search	Often offline or empirically fixed	Prone to premature convergence in high-density environments	Needs additional refinement to ensure AGV kinematics
Proposed algorithm	Bi-level PSO-guided adaptive continuous sampling	Online dynamic optimization driven by algorithm status	Robust failure-driven adaptive escape mechanism	Range-jitter-constrained B-splines ensuring strict kinematic feasibility

**Table 2:** List of PSO Parameters.

Index	Parameter Symbol	Description	Range
1	S	Base expansion step size	[0.5, 1.5]
2	$N_{stage1}$	Local exploration failure threshold	[3, 8]
3	$N_{stage2}$	Lateral bypass failure threshold	[8, 15]
4	κ	Base invalid-radius coefficient	[0.2, 0.7]
5	η	Density penalty intensity	[3, 8]

**Table 3:** Scenario A: Simulation data in a 22 × 22 sparse obstacle scenario. The *p*-values are calculated using the non-parametric Wilcoxon rank-sum test, comparing the search time of each baseline against the proposed algorithm.

Algorithm	Search Time	Sampling Nodes	Path Length	*p*-Value
Proposed algorithm	230.4697 ± 23.73	589 ± 61.8	147.1443 ± 7.54	-
Single offline PSO-optimized Bi-RRT	658.9588 ± 134.09	1589 ± 235.1	152.6167 ± 14.82	<0.01
Improved Bi-RRT without PSO	382.0215 ± 63.26	945 ± 136	149.9726 ± 14.21	<0.01
RRT* algorithm	292.462 ± 60.82	2700 ± 403.6	139.4076 ± 12.96	<0.01
RRT-Connect algorithm	260.3 ± 42.89	619 ± 96.1	155.9565 ± 18.01	<0.01
Bi-RRT algorithm	3170.6061 ± 564.21	1807 ± 284.1	199.4512 ± 20.39	<0.01
APF-Bi-RRT algorithm	575.5284 ± 226.41	781 ± 197.9	165.3734 ± 34.04	<0.01
GWO-Bi-RRT algorithm	148.8917 ± 54.82	1104 ± 277.3	162.519 ± 38.51	<0.01

**Table 4:** Performance comparison of different algorithms on the 10 planning instances in Scenario A: 22 × 22. Proposed: Proposed algorithm. PSO-Bi-RRT: Single offline PSO-optimized Bi-RRT. Bi-RRT-NoPSO: Improved Bi-RRT without PSO. Time: Search Time. Node: Sampling Node. Length: Path Length. Bold values indicate the results of the proposed algorithm.

Algorithm	Parameter	Proposed	PSO-Bi-RRT(*p*-Value)	Bi-RRT-NoPSO(*p*-Value)	RRT*(*p*-Value)	RRT-Connect(*p*-Value)	Bi-RRT(*p*-Value)	APF-Bi-RRT(*p*-Value)	GWO-Bi-RRT(*p*-Value)
Start→Target1	Time	**16.62 ± 1.99**	38.06 ± 9.19(<0.01)	48.62 ± 9.16(<0.01)	36.38 ± 8.83(<0.01)	33.50 ± 6.10(<0.01)	266.89 ± 54.82(<0.01)	79.81 ± 33.21(<0.01)	25.60 ± 10.06(<0.01)
	Node	**72.3 ± 9.4**	229.4 ± 42.2(<0.01)	115.4 ± 24.5(<0.01)	334.6 ± 64.5(<0.01)	72.1 ± 15.0(0.77)	206.8 ± 38.2(<0.01)	100.8 ± 29.2(<0.01)	130.4 ± 38.0(<0.01)
	Length	**18.18 ± 1.35**	18.36 ± 2.39(<0.01)	18.40 ± 2.48(0.57)	17.37 ± 2.28(0.97)	19.89 ± 3.26(<0.01)	22.71 ± 3.22(<0.01)	19.12 ± 4.59(0.20)	18.52 ± 5.20(0.93)
Start→Target2	Time	**35.85 ± 4.22**	104.92 ± 22.59(<0.01)	27.36 ± 5.31(<0.01)	32.19 ± 7.64(0.02)	77.95 ± 16.06(<0.01)	558.24 ± 104.04(<0.01)	104.54 ± 41.56(<0.01)	20.89 ± 8.35(<0.01)
	Node	**82.6 ± 10.0**	206.6 ± 42.1(<0.01)	107.3 ± 21.2(<0.01)	416.4 ± 82.9(<0.01)	85.4 ± 17.0(0.47)	278.4 ± 54.9(<0.01)	96.9 ± 28.6(<0.01)	160.0 ± 44.0(<0.01)
	Length	**23.62 ± 1.88**	22.82 ± 3.63(0.07)	23.58 ± 3.42(0.96)	22.26 ± 3.61(<0.01)	23.13 ± 3.52(0.58)	29.33 ± 5.36(<0.01)	23.03 ± 6.21(0.18)	22.50 ± 5.96(0.46)
Start→Target3	Time	**43.70 ± 5.26**	282.71 ± 65.97(<0.01)	38.05 ± 7.76(<0.01)	31.12 ± 7.44(<0.01)	34.99 ± 6.42(<0.01)	337.42 ± 79.30(<0.01)	54.61 ± 23.30(<0.01)	15.27 ± 5.84(<0.01)
	Node	**57.9 ± 7.0**	211.3 ± 36.0(<0.01)	74.7 ± 14.1(<0.01)	312.2 ± 55.3(<0.01)	74.5 ± 15.5(<0.01)	250.2 ± 47.4(<0.01)	98.7 ± 29.0(<0.01)	134.9 ± 39.0(<0.01)
	Length	**17.22 ± 1.38**	19.05 ± 3.04(<0.01)	17.14 ± 2.54(0.30)	15.70 ± 2.50(<0.01)	19.25 ± 3.46(<0.01)	28.04 ± 3.94(<0.01)	22.09 ± 4.88(<0.01)	22.42 ± 5.79(<0.01)
Start→Target4	Time	**13.64 ± 1.61**	12.64 ± 2.90(0.02)	26.85 ± 5.75(<0.01)	34.74 ± 9.00(<0.01)	34.74 ± 9.00(<0.01)	65.13 ± 13.75(<0.01)	21.14 ± 8.88(<0.01)	5.65 ± 2.26(<0.01)
	Node	**54.3 ± 7.6**	143.8 ± 27.3(<0.01)	92.2 ± 16.3(<0.01)	223.1 ± 45.8(<0.01)	47.2 ± 9.2(<0.01)	136.6 ± 24.6(<0.01)	55.0 ± 16.3(0.76)	78.8 ± 22.7(<0.01)
	Length	**12.07 ± 1.00**	11.62 ± 1.80(0.07)	12.31 ± 2.10(0.67)	11.74 ± 1.72(0.11)	11.99 ± 1.91(0.84)	15.49 ± 2.58(<0.01)	12.39 ± 2.80(0.39)	12.13 ± 3.74(0.67)
Target1→Target2	Time	**1.00 ± 0.0** **0**	1.00 ± 0.00	1.00 ± 0.00	1.00 ± 0.00	1.00 ± 0.00	1.00 ± 0.00	1.00 ± 0.00	1.00 ± 0.00
	Node	**60.0 ± 0.0**	60.0 ± 0.0	60.0 ± 0.0	60.0 ± 0.0	60.0 ± 0.0	60.0 ± 0.0	60.0 ± 0.0	60.0 ± 0.0
	Length	**6.00 ± 0.00**	6.00 ± 0.00	6.00 ± 0.00	6.00 ± 0.00	6.00 ± 0.00	6.00 ± 0.00	6.00 ± 0.00	6.00 ± 0.00
Target1→Target3	Time	**14.69 ± 1.92**	15.03 ± 3.90(0.44)	33.15 ± 5.99(<0.01)	27.17 ± 7.22(<0.01)	10.77 ± 1.81(<0.01)	317.72 ± 63.79(<0.01)	49.00 ± 18.71(<0.01)	15.53 ± 6.18(0.12)
	Node	**40.4 ± 4.9**	94.2 ± 17.9(<0.01)	68.4 ± 12.4(<0.01)	230.4 ± 41.7(<0.01)	42.0 ± 8.8(0.48)	161.5 ± 30.1(<0.01)	69.2 ± 21.0(<0.01)	112.9 ± 31.7(<0.01)
	Length	**9.24 ± 0.70**	8.86 ± 1.28(0.06)	9.41 ± 1.45(0.7)	8.92 ± 1.27(0.07)	12.05 ± 2.09(<0.01)	16.33 ± 2.21(<0.01)	12.82 ± 2.74(<0.01)	15.73 ± 4.55(<0.01)
Target1→Target4	Time	**39.70 ± 4.41**	33.36 ± 7.78(<0.01)	64.05 ± 11.18(<0.01)	35.46 ± 7.55(<0.01)	19.13 ± 3.93(<0.01)	547.12 ± 118.29(<0.01)	52.53 ± 22.99(<0.01)	16.70 ± 6.38(<0.01)
	Node	**57.1 ± 8.2**	175.1 ± 34.1(<0.01)	104.9 ± 20.0(<0.01)	282.5 ± 53.6(<0.01)	54.8 ± 11.4(0.10)	162.2 ± 30.2(<0.01)	62.4 ± 17.9(0.21)	96.0 ± 28.5(<0.01)
	Length	**14.16 ± 0.93**	14.12 ± 2.15(0.92)	13.75 ± 1.86(0.11)	12.91 ± 2.08(<0.01)	13.93 ± 2.15(0.47)	17.88 ± 2.86(<0.01)	14.78 ± 3.50(0.27)	13.77 ± 3.91(0.49)
Target2→Target3	Time	**21.35 ± 2.40**	54.15 ± 13.13(<0.01)	15.86 ± 3.55(<0.01)	29.25 ± 6.32(<0.01)	19.24 ± 3.75(<0.01)	413.36 ± 75.58(<0.01)	58.03 ± 24.76(<0.01)	14.23 ± 5.42(<0.01)
	Node	**65.5 ± 8.4**	157.0 ± 32.1(<0.01)	81.2 ± 14.6(<0.01)	297.6 ± 50.9(<0.01)	60.0 ± 12.0(<0.01)	178.1 ± 37.9(<0.01)	87.8 ± 28.0(<0.01)	106.3 ± 31.6(<0.01)
	Length	**14.69 ± 1.16**	21.25 ± 2.76(<0.01)	14.74 ± 1.97(0.99)	14.43 ± 1.84(0.13)	16.71 ± 2.22(<0.01)	20.90 ± 2.87(<0.01)	20.32 ± 4.74(<0.01)	16.28 ± 3.84(0.01)
Target2→Target4	Time	**13.90 ± 1.38**	85.06 ± 18.30(<0.01)	82.00 ± 16.48(<0.01)	35.04 ± 7.42(<0.01)	38.27 ± 7.75(<0.01)	252.91 ± 59.89(<0.01)	96.08 ± 38.19(<0.01)	18.07 ± 6.90(<0.01)
	Node	**43.1 ± 5.3**	136.7 ± 27.7(<0.01)	124.8 ± 22.3(<0.01)	238.3 ± 44.2(<0.01)	58.8 ± 12.0(<0.01)	153.8 ± 30.1(<0.01)	74.0 ± 22.2(<0.01)	111.9 ± 33.7(<0.01)
	Length	**14.06 ± 1.06**	13.03 ± 2.09(<0.01)	16.88 ± 2.66(<0.01)	13.15 ± 1.97(<0.01)	15.45 ± 2.33(<0.01)	19.21 ± 2.73(<0.01)	17.00 ± 4.36(<0.01)	17.84 ± 4.47(<0.01)
Target3→Target4	Time	**30.01 ± 3.52**	32.04 ± 8.02(0.16)	45.08 ± 8.75(<0.01)	30.11 ± 7.15(0.74)	17.86 ± 3.30(<0.01)	410.82 ± 85.28(<0.01)	58.78 ± 24.40(<0.01)	15.96 ± 6.15(<0.01)
	Node	**55.7 ± 7.5**	175.1 ± 28.7(<0.01)	116.1 ± 21.5(<0.01)	304.9 ± 55.3(<0.01)	64.1 ± 14.8(<0.01)	219.3 ± 47.0(<0.01)	76.2 ± 22.9(<0.01)	113.0 ± 33.0(<0.01)
	Length	**17.91 ± 1.26**	17.51 ± 2.61(0.11)	17.77 ± 2.78(0.40)	16.94 ± 2.50(0.03)	17.57 ± 3.01(0.25)	23.56 ± 3.45(<0.01)	17.83 ± 4.36(0.82)	17.34 ± 4.62(0.34)

**Table 5:** Scenario A: Simulation data in a 33 × 33 medium obstacle scenario. The *p*-values are calculated using the non-parametric Wilcoxon rank-sum test, comparing the search time of each baseline against the proposed algorithm.

Algorithm	Search Time	Sampling Nodes	Path Length	*p*-Value
Proposed algorithm	298.3399 ± 38.96	856 ± 87.1	238.5176 ± 12.18	-
Single offline PSO-optimized Bi-RRT	581.9477 ± 134.92	1631 ± 261	233.3437 ± 23.91	<0.01
Improved Bi-RRT without PSO	459.6197 ± 100.95	1058 ± 170	271.0580 ± 24.19	<0.01
RRT* algorithm	353.1700 ± 101.7	2700 ± 463.5	248.7351 ± 24.56	<0.01
RRT-Connect algorithm	328.6018 ± 79.78	930 ± 132.5	255.4169 ± 28.04	<0.01
Bi-RRT algorithm	7701.7133 ± 1612.29	1818 ± 269.5	294.0391 ± 33.93	<0.01
APF-Bi-RRT algorithm	1923.3931 ± 819.79	1283 ± 312.4	283.59 ± 57.03	<0.01
GWO-Bi-RRT algorithm	350.535 ± 116.59	1383 ± 327.5	278.8904 ± 54.42	<0.01

**Table 6:** Performance comparison of different algorithms on the 10 planning instances in Scenario A: 33 × 33. Proposed: Proposed algorithm. PSO-Bi-RRT: Single offline PSO-optimized Bi-RRT. Bi-RRT-NoPSO: Improved Bi-RRT without PSO. Time: Search Time. Node: Sampling Node. Length: Path Length. Bold values indicate the results of the proposed algorithm.

Algorithm	Parameter	Proposed	PSO-Bi-RRT(*p*-Value)	Bi-RRT-NoPSO(*p*-Value)	RRT*(*p*-Value)	RRT-Connect(*p*-Value)	Bi-RRT(*p*-Value)	APF-Bi-RRT(*p*-Value)	GWO-Bi-RRT(*p*-Value)
Start→Target1	Time	**22.58 ± 2.89**	38.33 ± 10.76(<0.01)	72.72 ± 18.58(<0.01)	48.92 ± 14.71(<0.01)	48.09 ± 12.91(<0.01)	702.88 ± 174.64(<0.01)	317.63 ± 141.94(<0.01)	68.32 ± 25.03(<0.01)
	Node	**86.6 ± 11.1**	198.5 ± 36.9(<0.01)	109.3 ± 23.7(<0.01)	187.0 ± 40.2(<0.01)	87.6 ± 17.6(0.69)	197.1 ± 38.2(<0.01)	130.3 ± 38.4(<0.01)	133.7 ± 36.1(<0.01)
	Length	**31.04 ± 2.17**	29.25 ± 4.19(<0.01)	35.91 ± 5.13(<0.01)	32.68 ± 5.13(0.02)	34.59 ± 5.23(<0.01)	35.31 ± 6.16(<0.01)	35.09 ± 7.59(<0.01)	34.32 ± 7.54(0.02)
Start→Target2	Time	**48.70 ± 7.69**	105.66 ± 26.02(<0.01)	40.92 ± 9.83(<0.01)	43.29 ± 13.80(<0.01)	111.90 ± 28.97(<0.01)	1470.19 ± 324.49(<0.01)	416.06 ± 183.17(<0.01)	55.76 ± 20.03(0.01)
	Node	**99.1 ± 12.8**	178.9 ± 37.5(<0.01)	101.7 ± 21.1(0.83)	232.7 ± 49.1(<0.01)	103.7 ± 19.3(0.17)	265.3 ± 48.2(<0.01)	125.3 ± 35.0(<0.01)	164.1 ± 48.0(<0.01)
	Length	**40.34 ± 3.25**	36.34 ± 5.33(<0.01)	46.01 ± 6.26(<0.01)	41.88 ± 5.43(0.11)	40.23 ± 6.29(0.92)	45.60 ± 7.01(<0.01)	42.29 ± 10.03(0.24)	41.69 ± 9.48(0.57)
Start→Target3	Time	**59.36 ± 7.57**	284.71 ± 67.26(<0.01)	56.91 ± 12.83(0.54)	41.85 ± 12.61(<0.01)	50.23 ± 13.22(<0.01)	888.64 ± 206.16(<0.01)	217.36 ± 93.66(<0.01)	40.76 ± 14.97(<0.01)
	Node	**69.5 ± 8.8**	182.8 ± 35.2(<0.01)	70.7 ± 14.0(0.91)	174.4 ± 37.9(<0.01)	90.5 ± 19.3(<0.01)	238.4 ± 43.9(<0.01)	127.5 ± 38.1(<0.01)	138.4 ± 40.5(<0.01)
	Length	**29.41 ± 2.08**	30.34 ± 4.34(0.09)	33.45 ± 4.80(<0.01)	29.53 ± 4.66(0.99)	33.48 ± 5.37(<0.01)	43.59 ± 7.83(<0.01)	40.55 ± 9.95(<0.01)	41.54 ± 10.20(<0.01)
Start→Target4	Time	**18.53 ± 2.68**	12.73 ± 3.41(<0.01)	40.17 ± 9.71(<0.01)	46.72 ± 13.54(<0.01)	10.90 ± 2.75(<0.01)	171.54 ± 40.72(<0.01)	84.13 ± 37.82(<0.01)	15.07 ± 5.23(<0.01)
	Node	**65.2 ± 8.7**	124.4 ± 23.9(<0.01)	87.4 ± 17.7(<0.01)	124.7 ± 27.0(<0.01)	57.4 ± 12.5(<0.01)	130.3 ± 28.4(<0.01)	70.9 ± 19.8(0.12)	80.7 ± 23.3(<0.01)
	Length	**20.61 ± 1.54**	18.51 ± 3.10(<0.01)	24.02 ± 4.27(<0.01)	22.08 ± 3.19(0.02)	20.85 ± 3.49(0.95)	24.08 ± 3.86(<0.01)	22.74 ± 5.41(0.06)	22.47 ± 5.07(0.02)
Target1→Target2	Time	**4.52 ± 0.68**	4.00 ± 1.16(<0.01)	11.39 ± 2.60(<0.01)	7.35 ± 2.29(<0.01)	10.29 ± 2.91(<0.01)	19.25 ± 4.46(<0.01)	18.21 ± 7.70(<0.01)	3.02 ± 1.02(<0.01)
	Node	**161.4 ± 19.7**	313.8 ± 66.5(<0.01)	225.9 ± 44.0(<0.01)	1246.3 ± 229.0(<0.01)	210.2 ± 34.2(<0.01)	187.8 ± 34.5(<0.01)	335.4 ± 94.4(<0.01)	314.7 ± 82.5(<0.01)
	Length	**10.30 ± 0.81**	9.38 ± 1.66(<0.01)	11.86 ± 1.77(<0.01)	11.30 ± 1.76(<0.01)	10.30 ± 1.82(0.98)	11.98 ± 1.75(<0.01)	11.05 ± 2.61(0.04)	10.74 ± 2.24(0.47)
Target1→Target3	Time	**19.96 ± 2.98**	15.13 ± 4.23(<0.01)	49.58 ± 12.46(<0.01)	36.54 ± 11.86(<0.01)	15.46 ± 4.35(<0.01)	836.76 ± 199.07(<0.01)	195.01 ± 87.74(<0.01)	41.44 ± 13.87(<0.01)
	Node	**48.4 ± 6.0**	81.6 ± 18.0(<0.01)	64.9 ± 13.2(<0.01)	128.6 ± 30.9(<0.01)	51.1 ± 11.2(0.19)	153.9 ± 27.6(<0.01)	89.3 ± 26.7(<0.01)	115.9 ± 32.7(<0.01)
	Length	**15.78 ± 1.17**	14.11 ± 2.16(<0.01)	18.36 ± 2.58(<0.01)	16.78 ± 2.82(0.23	20.95 ± 3.38(<0.01)	25.39 ± 4.02(<0.01)	23.53 ± 5.49(<0.01)	29.15 ± 7.37(<0.01)
Target1→Target4	Time	**53.92 ± 7.90**	33.60 ± 8.98(<0.01)	95.79 ± 24.10(<0.01)	47.68 ± 15.26(0.01)	27.47 ± 7.16(<0.01)	1440.90 ± 347.44(<0.01)	209.07 ± 91.94(<0.01)	44.58 ± 16.21(<0.01)
	Node	**68.4 ± 9.7**	151.6 ± 30.7(<0.01)	99.4 ± 22.0(<0.01)	157.8 ± 35.6(<0.01)	66.6 ± 14.6(0.54)	154.5 ± 33.8(<0.01)	80.5 ± 22.5(<0.01)	98.5 ± 28.5(<0.01)
	Length	**24.18 ± 1.58**	22.50 ± 3.21(<0.01)	26.83 ± 3.83(<0.01)	24.29 ± 3.90(0.87)	24.23 ± 3.86(0.34)	27.80 ± 4.40(<0.01)	27.14 ± 6.13(<0.01)	25.51 ± 5.55(0.24)
Target2→Target3	Time	**29.00 ± 4.09**	54.53 ± 14.80(<0.01)	23.72 ± 5.55(<0.01)	39.33 ± 12.01(<0.01)	27.62 ± 7.19(0.21)	1088.63 ± 244.16(<0.01)	230.96 ± 98.33(<0.01)	37.98 ± 13.26(<0.01)
	Node	**78.6 ± 10.3**	135.9 ± 26.6(<0.01)	76.9 ± 17.1(0.26)	166.2 ± 36.1(<0.01)	72.9 ± 13.5(0.02)	169.7 ± 30.9(<0.01)	113.5 ± 33.4(<0.01)	109.1 ± 31.3(<0.01)
	Length	**25.08 ± 2.23**	33.84 ± 5.96(<0.01)	28.76 ± 4.43(<0.01)	27.14 ± 4.11(0.02	29.06 ± 4.71(<0.01)	32.49 ± 5.33(<0.01)	37.31 ± 8.75(<0.01)	30.17 ± 6.44(<0.01)
Target2→Target4	Time	**1.00 ± 0.00**	1.00 ± 0.00	1.00 ± 0.00	1.00 ± 0.00	1.00 ± 0.00	1.00 ± 0.00	1.00 ± 0.00	1.00 ± 0.00
	Node	**112.0 ± 0.0**	112.0 ± 0.0	112.0 ± 0.0	112.0 ± 0.0	112.0 ± 0.0	112.0 ± 0.0	112.0 ± 0.0	112.0 ± 0.0
	Length	**11.18 ± 0.00**	11.18 ± 0.00	11.18 ± 0.00	11.18 ± 0.00	11.18 ± 0.00	11.18 ± 0.00	11.18 ± 0.00	11.18 ± 0.00
Target3→Target4	Time	**40.76 ± 5.71**	32.26 ± 9.33(<0.01)	67.43 ± 16.57(<0.01)	40.49 ± 13.09(0.98)	25.63 ± 6.79(<0.01)	1081.93 ± 271.83(<0.01)	233.96 ± 103.33(<0.01)	42.59 ± 13.48(0.33)
	Node	**66.7 ± 8.9**	151.6 ± 29.2(<0.01)	109.9 ± 22.9(<0.01)	170.3 ± 43.5(<0.01)	77.9 ± 15.9(<0.01)	208.9 ± 42.3(<0.01)	98.4 ± 29.3(<0.01)	115.9 ± 32.2(<0.01)
	Length	**30.59 ± 2.27**	27.89 ± 4.10(<0.01)	34.68 ± 5.14(<0.01)	31.86 ± 4.68(0.13)	30.55 ± 4.31(0.90)	36.62 ± 6.01(<0.01)	32.72 ± 8.10(0.12)	32.13 ± 7.82(0.38)

**Table 7:** Scenario A: Simulation data in a 44 × 44 dense obstacle scenario. The *p*-values are calculated using the non-parametric Wilcoxon rank-sum test, comparing the search time of each baseline against the proposed algorithm.

Algorithm	Search Time	Sampling Nodes	Path Length	*p*-Value
Proposed algorithm	180.1058 ± 31.18	915 ± 144.0	185.1485 ± 19.51	-
Single offline PSO-optimized Bi-RRT	3101.8616 ± 814.48	1912 ± 369.0	438.6135 ± 54.89	<0.01
Improved Bi-RRT without PSO	537.3699 ± 160.41	1512 ± 286.6	422.972 ± 58.96	<0.01
RRT* algorithm	479.0341 ± 143.06	5000 ± 996.4	391.3012 ± 49.75	<0.01
RRT-Connect algorithm	2516.0623 ± 697.73	2074 ± 431.9	441.4096 ± 61.85	<0.01
Bi-RRT algorithm	7902.213 ± 2488.67	2270 ± 417.9	484.0086 ± 78.07	<0.01
APF-Bi-RRT algorithm	2118.8465 ± 775.48	1899 ± 510.8	461.0714 ± 77.79	<0.01
GWO-Bi-RRT algorithm	212.7069 ± 82.47	1348 ± 338.2	215.1534 ± 36.47	<0.01

**Table 8:** Performance comparison of different algorithms on the 10 planning instances in Scenario A: 44 × 44. Bold values indicate the results of the proposed algorithm.

Algorithm	Parameter	Proposed	PSO-Bi-RRT(*p*-Value)	Bi-RRT-NoPSO(*p*-Value)	RRT*(*p*-Value)	RRT-Connect(*p*-Value)	Bi-RRT(*p*-Value)	APF-Bi-RRT(*p*-Value)	GWO-Bi-RRT(*p*-Value)
Start→Target1	Time	**12.86 ± 2.24**	178.36 ± 50.17(<0.01)	67.22 ± 22.25(<0.01)	58.69 ± 18.29(<0.01)	316.30 ± 85.33(<0.01)	663.85 ± 209.03(<0.01)	292.01 ± 112.05(<0.01)	36.54 ± 14.68(<0.01)
	Node	**99.8 ± 15.5**	232.0 ± 48.8(<0.01)	155.1 ± 29.4(<0.01)	343.5 ± 80.7(<0.01)	204.2 ± 45.9(<0.01)	241.4 ± 49.2(<0.01)	195.2 ± 59.8(<0.01)	130.1 ± 38.1(<0.01)
	Length	**22.87 ± 2.65**	52.81 ± 8.11(<0.01)	51.87 ± 8.67(<0.01)	48.75 ± 8.36(<0.01)	56.32 ± 8.17(<0.01)	54.64 ± 9.91(<0.01)	53.29 ± 11.35(<0.01)	24.55 ± 4.20(0.04)
Start→Target2	Time	**27.74 ± 4.91**	491.65 ± 146.46(<0.01)	37.83 ± 12.70(<0.01)	51.93 ± 16.95(<0.01)	736.02 ± 217.01(<0.01)	1388.56 ± 477.19(<0.01)	382.49 ± 154.03(<0.01)	29.82 ± 12.10(0.18)
	Node	**113.9 ± 18.0**	208.8 ± 47.1(<0.01)	144.4 ± 32.8(<0.01)	427.5 ± 99.9(<0.01)	241.8 ± 64.0(<0.01)	325.0 ± 68.5(<0.01)	187.8 ± 56.1(<0.01)	159.6 ± 46.0(<0.01)
	Length	**29.72 ± 3.41**	65.62 ± 10.78(<0.01)	66.46 ± 12.46(<0.01)	62.48 ± 10.68(<0.01)	65.50 ± 10.72(<0.01)	70.57 ± 13.34(<0.01)	64.21 ± 12.52(<0.01)	29.82 ± 6.33(0.41)
Start→Target3	Time	**33.81 ± 6.35**	1324.81 ± 362.53(<0.01)	52.61 ± 17.39(<0.01)	50.21 ± 17.27(<0.01)	330.39 ± 93.59(<0.01)	839.30 ± 287.41(<0.01)	199.83 ± 76.08(<0.01)	21.80 ± 8.72(<0.01)
	Node	**79.8 ± 14.1**	213.5 ± 48.4(<0.01)	100.6 ± 21.4(<0.01)	320.5 ± 80.8(<0.01)	210.9 ± 46.2(<0.01)	292.2 ± 58.2(<0.01)	191.0 ± 55.2(<0.01)	134.6 ± 39.0(<0.01)
	Length	**21.66 ± 2.68**	54.79 ± 9.00(<0.01)	48.31 ± 7.55(<0.01)	44.06 ± 7.82(<0.01)	54.51 ± 11.37(<0.01)	67.47 ± 13.52(<0.01)	61.59 ± 11.14(<0.01)	29.72 ± 6.08(<0.01)
Start→Target4	Time	**10.56 ± 1.80**	59.25 ± 17.25(<0.01)	37.13 ± 12.74(<0.01)	56.05 ± 17.10(<0.01)	71.71 ± 21.55(<0.01)	162.01 ± 54.38(<0.01)	77.35 ± 28.83(<0.01)	8.06 ± 3.30(<0.01)
	Node	**74.9 ± 12.8**	145.3 ± 33.1(<0.01)	124.1 ± 29.9(<0.01)	229.0 ± 57.2(<0.01)	134.0 ± 30.8(<0.01)	159.7 ± 32.4(<0.01)	106.4 ± 31.1(<0.01)	78.6 ± 22.0(0.39)
	Length	**15.18 ± 1.63**	33.43 ± 6.09(<0.01)	34.69 ± 5.57(<0.01)	32.94 ± 5.22(<0.01)	33.94 ± 6.65(<0.01)	37.27 ± 7.02(<0.01)	34.53 ± 6.79(<0.01)	16.07 ± 3.61(0.37)
Target1→Target2	Time	**2.58 ± 0.49**	18.61 ± 5.31(<0.01)	10.53 ± 3.39(<0.01)	8.82 ± 2.86(<0.01)	67.69 ± 20.04(<0.01)	18.18 ± 5.80(<0.01)	16.74 ± 6.32(<0.01)	1.62 ± 0.63(<0.01)
	Node	**185.7 ± 31.3**	366.3 ± 82.5(<0.01)	321.0 ± 71.2(<0.01)	2290.0 ± 470.5(<0.01)	490.2 ± 109.7(<0.01)	230.1 ± 52.4(<0.01)	502.9 ± 135.2(<0.01)	306.0 ± 79.5(<0.01)
	Length	**7.59 ± 0.92**	16.93 ± 3.14(<0.01)	17.13 ± 2.96(<0.01)	16.86 ± 2.76(<0.01)	16.77 ± 2.72(<0.01)	18.54 ± 3.55(<0.01)	16.78 ± 3.57(<0.01)	7.68 ± 1.68(0.73)
Target1→Target3	Time	**11.37 ± 2.08**	70.41 ± 21.12(<0.01)	45.84 ± 15.15(<0.01)	43.84 ± 13.21(<0.01)	101.66 ± 31.98(<0.01)	790.30 ± 265.00(<0.01)	179.27 ± 69.71(<0.01)	22.16 ± 8.81(<0.01)
	Node	**55.7 ± 9.4**	95.2 ± 20.0(<0.01)	92.0 ± 20.3(<0.01)	236.5 ± 58.1(<0.01)	119.2 ± 29.4(<0.01)	188.7 ± 40.2(<0.01)	134.0 ± 41.5(<0.01)	112.8 ± 31.9(<0.01)
	Length	**11.63 ± 1.49**	25.49 ± 4.18(<0.01)	26.53 ± 5.17(<0.01)	25.04 ± 4.62(<0.01)	34.12 ± 6.61(<0.01)	39.30 ± 7.88(<0.01)	35.73 ± 8.14(<0.01)	20.85 ± 4.26(<0.01)
Target1→Target4	Time	**30.71 ± 5.48**	156.33 ± 45.10(<0.01)	88.56 ± 27.64(<0.01)	57.20 ± 17.71(<0.01)	180.67 ± 48.93(<0.01)	1360.90 ± 444.02(<0.01)	192.20 ± 76.15(<0.01)	23.84 ± 9.68(<0.01)
	Node	**78.7 ± 13.6**	177.0 ± 37.2(<0.01)	141.3 ± 29.6(<0.01)	290.0 ± 65.5(<0.01)	155.3 ± 38.0(<0.01)	189.3 ± 40.0(<0.01)	120.6 ± 33.8(<0.01)	95.8 ± 26.2(<0.01)
	Length	**17.81 ± 2.15**	40.62 ± 6.68(<0.01)	38.75 ± 7.35(<0.01)	36.23 ± 6.05(<0.01)	39.46 ± 7.17(<0.01)	43.03 ± 8.33(<0.01)	41.21 ± 7.85(<0.01)	18.25 ± 4.03(0.79)
Target2→Target3	Time	**16.52 ± 3.07**	253.73 ± 68.31(<0.01)	21.92 ± 6.46(<0.01)	47.19 ± 15.05(<0.01)	181.68 ± 59.08(<0.01)	1028.18 ± 317.27(<0.01)	212.33 ± 78.57(<0.01)	20.31 ± 8.13(<0.01)
	Node	**90.4 ± 15.0**	158.7 ± 37.2(<0.01)	109.2 ± 24.7(<0.01)	305.6 ± 72.7(<0.01)	170.1 ± 39.0(<0.01)	208.0 ± 45.4(<0.01)	170.1 ± 52.0(<0.01)	106.1 ± 27.9(<0.01)
	Length	**18.48 ± 2.44**	61.10 ± 10.31(<0.01)	41.54 ± 7.19(<0.01)	40.50 ± 7.37(<0.01)	47.31 ± 8.21(<0.01)	50.29 ± 10.81(<0.01)	56.65 ± 11.40(<0.01)	21.58 ± 3.83(<0.01)
Target2→Target4	Time	**10.75 ± 1.95**	398.59 ± 115.77(<0.01)	113.39 ± 34.44(<0.01)	56.53 ± 18.41(<0.01)	361.34 ± 115.29(<0.01)	629.08 ± 219.21(<0.01)	351.55 ± 127.33(<0.01)	25.80 ± 10.86(<0.01)
	Node	**59.4 ± 9.2**	138.0 ± 29.1(<0.01)	168.0 ± 35.9(<0.01)	244.4 ± 56.2(<0.01)	166.7 ± 43.2(<0.01)	179.5 ± 44.3(<0.01)	143.4 ± 45.7(<0.01)	111.8 ± 28.3(<0.01)
	Length	**17.68 ± 1.94**	37.46 ± 6.71(<0.01)	47.59 ± 7.83(<0.01)	36.90 ± 5.60(<0.01)	43.74 ± 7.83(<0.01)	46.22 ± 9.04(<0.01)	47.39 ± 9.85(<0.01)	23.65 ± 4.59(<0.01)
Target3→Target4	Time	**23.22 ± 4.46**	150.13 ± 45.08(<0.01)	62.33 ± 19.52(<0.01)	48.58 ± 15.86(<0.01)	168.59 ± 49.43(<0.01)	1021.86 ± 357.01(<0.01)	215.08 ± 80.95(<0.01)	22.78 ± 9.12(0.75)
	Node	**76.7 ± 13.8**	177.1 ± 40.7(<0.01)	156.2 ± 33.6(<0.01)	312.9 ± 74.3(<0.01)	181.5 ± 39.3(<0.01)	256.0 ± 54.4(<0.01)	147.6 ± 42.8(<0.01)	112.6 ± 28.8(<0.01)
	Length	**22.53 ± 2.66**	50.36 ± 8.06(<0.01)	50.09 ± 9.27(<0.01)	47.54 ± 8.06(<0.01)	49.74 ± 8.60(<0.01)	56.68 ± 10.45(<0.01)	49.69 ± 9.46(<0.01)	22.98 ± 4.68(0.90)

**Table 9:** Scenario B: Simulation data in a narrow passage scenario. The *p*-values are calculated using the non-parametric Wilcoxon rank-sum test, comparing the search time of each baseline against the proposed algorithm.

Algorithm	Search Time	Sampling Nodes	Path Length	*p*-Value
Proposed algorithm	20.7136 ± 4.58	134 ± 21.2	142.9661 ± 12.79	-
Single offline PSO-optimized Bi-RRT	426.1561 ± 200.99	581.156 ± 155.4	171.4162 ± 35.08	<0.01
Improved Bi-RRT without PSO	39.245 ± 13.31	191 ± 48.8	143.4612 ± 35.23	<0.01
RRT* algorithm	25.8061 ± 11.3	10,000 ± 2486.3	142.3402 ± 28.12	<0.01
RRT-Connect algorithm	464.7506 ± 211.23	595.6 ± 137.7	145.2165 ± 25.77	<0.01
Bi-RRT algorithm	462.3854 ± 192.65	573.6667 ± 141.5	185.403 ± 37.59	<0.01
APF-Bi-RRT algorithm	232.3129 ± 93.13	307.2 ± 84.8	143.7397 ± 26.73	<0.01
GWO-Bi-RRT algorithm	582.5142 ± 246.25	738.5 ± 183.3	143.0092 ± 26.19	<0.01

**Table 10:** Scenario C: Simulation data in a U-shaped obstacle scenario. The *p*-values are calculated using the non-parametric Wilcoxon rank-sum test, comparing the search time of each baseline against the proposed algorithm.

Algorithm	Search Time	Sampling Nodes	Path Length	*p*-Value
Proposed algorithm	17.4002 ± 3.47	342 ± 51.4	51.3306 ± 4.75	-
Single offline PSO-optimized Bi-RRT	351.9740 ± 138.45	485 ± 112	52.1463 ± 11.68	<0.01
Improved Bi-RRT without PSO	37.4002 ± 16.79	544 ± 152.6	52.5790 ± 10.61	<0.01
RRT* algorithm	20.5943 ± 9.12	10,000 ± 2910.2	49.6891 ± 10.48	0.1023
RRT-Connect algorithm	284.8062 ± 115.28	537 ± 148.5	57.0033 ± 10.82	<0.01
Bi-RRT algorithm	407.5826 ± 152.92	500 ± 114.8	53.5640 ± 10.26	<0.01
APF-Bi-RRT algorithm	573.6202 ± 226.81	385 ± 95.7	55.4062 ± 10.91	<0.01
GWO-Bi-RRT algorithm	332.8788 ± 89.85	435 ± 102.2	52.7478 ± 13.15	<0.01

## Data Availability

The original contributions presented in the study are included in the article. Further inquiries can be directed to the corresponding author.

## Supplementary Materials

The following supporting information can be downloaded at https://www.mdpi.com/article/10.3390/s26134062/s1. Table S1: Definitions of the planning instances and their corresponding supplementary figures. Figure S1: Comparative path planning results for Instance P1. (a) Proposed algorithm. (b) Single offline PSO-optimized Bi-RRT. (c) Improved Bi-RRT without PSO. (d) RRT* algorithm. (e) RRT-Connect algorithm. (f) Bi-RRT algorithm. (g) APF-Bi-RRT algorithm. (h) GWO-Bi-RRT algorithm. Figure S2: Comparative path planning results for Instance P2. (a) Proposed algorithm. (b) Single offline PSO-optimized Bi-RRT. (c) Improved Bi-RRT without PSO. (d) RRT* algorithm. (e) RRT-Connect algorithm. (f) Bi-RRT algorithm. (g) APF-Bi-RRT algorithm. (h) GWO-Bi-RRT algorithm. Figure S3: Comparative path planning results for Instance P3. (a) Proposed algorithm. (b) Single offline PSO-optimized Bi-RRT. (c) Improved Bi-RRT without PSO. (d) RRT* algorithm. (e) RRT-Connect algorithm. (f) Bi-RRT algorithm. (g) APF-Bi-RRT algorithm. (h) GWO-Bi-RRT algorithm. Figure S4: Comparative path planning results for Instance P4. (a) Proposed algorithm. (b) Single offline PSO-optimized Bi-RRT. (c) Improved Bi-RRT without PSO. (d) RRT* algorithm. (e) RRT-Connect algorithm. (f) Bi-RRT algorithm. (g) APF-Bi-RRT algorithm. (h) GWO-Bi-RRT algorithm. Figure S5: Comparative path planning results for Instance P5. (a) Proposed algorithm. (b) Single offline PSO-optimized Bi-RRT. (c) Improved Bi-RRT without PSO. (d) RRT* algorithm. (e) RRT-Connect algorithm. (f) Bi-RRT algorithm. (g) APF-Bi-RRT algorithm. (h) GWO-Bi-RRT algorithm. Figure S6: Comparative path planning results for Instance P6. (a) Proposed algorithm. (b) Single offline PSO-optimized Bi-RRT. (c) Improved Bi-RRT without PSO. (d) RRT* algorithm. (e) RRT-Connect algorithm. (f) Bi-RRT algorithm. (g) APF-Bi-RRT algorithm. (h) GWO-Bi-RRT algorithm. Figure S7: Comparative path planning results for Instance P7. (a) Proposed algorithm. (b) Single offline PSO-optimized Bi-RRT. (c) Improved Bi-RRT without PSO. (d) RRT* algorithm. (e) RRT-Connect algorithm. (f) Bi-RRT algorithm. (g) APF-Bi-RRT algorithm. (h) GWO-Bi-RRT algorithm. Figure S8: Comparative path planning results for Instance P8. (a) Proposed algorithm. (b) Single offline PSO-optimized Bi-RRT. (c) Improved Bi-RRT without PSO. (d) RRT* algorithm. (e) RRT-Connect algorithm. (f) Bi-RRT algorithm. (g) APF-Bi-RRT algorithm. (h) GWO-Bi-RRT algorithm. Figure S9: Comparative path planning results for Instance P9. (a) Proposed algorithm. (b) Single offline PSO-optimized Bi-RRT. (c) Improved Bi-RRT without PSO. (d) RRT* algorithm. (e) RRT-Connect algorithm. (f) Bi-RRT algorithm. (g) APF-Bi-RRT algorithm. (h) GWO-Bi-RRT algorithm.

## Abbreviations

The following abbreviations are used in this manuscript:

AGV	Automated Guided Vehicle
PSO	Particle Swarm Optimizations
Bi-RRT	Bidirectional Rapidly Exploring Random Tree
CLPSO	Comprehensive Learning Particle Swarm Optimization
CLS	Comprehensive Learning Strategy
RRT*	Rapidly Exploring Random Tree
RRT-Connect	Rapidly Exploring Random Tree Connect
PSO-Bi-RRT	Particle Swarm Optimization-Bidirectional Rapidly Exploring Random Tree
