# Supplementary material for: A Bi-Level Hybrid Framework for Multi-Target Path Planning of AGV Based on Particle Swarm Optimization and Bidirectional Rapidly Exploring Random Tree
Source: Sensors (Basel). 2026 Jun 26;26(13):4062. doi: 10.3390/s26134062 (PMC13364332; doi:10.3390/s26134062)
Supplement: Supplementary file 1 [file sensors-26-04062-s001.zip › sensors-4338773-supplementary.pdf]

To enhance the transparency and reproducibility of the experimental evaluation, this supplementary document provides detailed trajectory visualizations for the independent planning instances involved in a representative multi-target scenario presented in the main manuscript. These results complement the quantitative analyses reported in the experimental section and provide additional evidence regarding the effectiveness and robustness of the proposed framework.

Specifically, the 44 × 44 dense obstacle environment (Scenario A) was selected as a representative case study because it poses greater challenges to path planning due to its high obstacle density and environmental complexity. Pairwise path planning was performed among the predefined target nodes, resulting in multiple independent planning instances within this scenario. The definitions of these planning instances and their corresponding supplementary figures are summarized in Table S1.

For each planning instance, the path planning trajectories generated by all compared algorithms under identical environmental conditions are presented in the corresponding figures. These visualizations provide additional support for the comparative analyses reported in the manuscript and further demonstrate the effectiveness of the proposed framework across different planning tasks within a challenging environment.

**Table S1.** Definitions of the planning instances and their corresponding supplementary figures.

| Instance ID | Planning Task | Figures ID |
|---|---|---|
| P1 | Start→Target2 | Figure S1 |
| P2 | Start→Target3 | Figure S2 |
| P3 | Start→Target4 | Figure S3 |
| P4 | Target1→Target2 | Figure S4 |
| P5 | Target1→Target3 | Figure S5 |
| P6 | Target1→Target4 | Figure S6 |
| P7 | Target2→Target3 | Figure S7 |
| P8 | Target2→Target4 | Figure S8 |
| P9 | Target3→Target4 | Figure S9 |

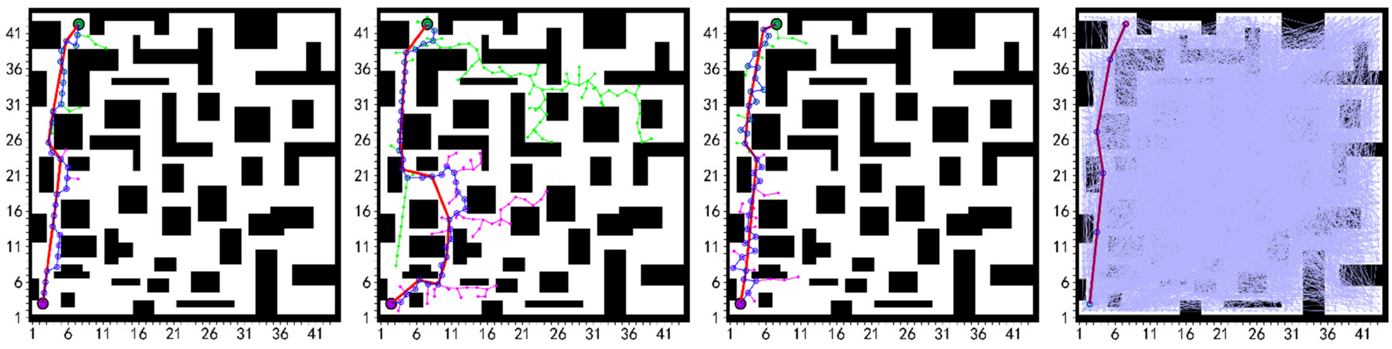

(**a**) (**b**) (**c**) (**d**)

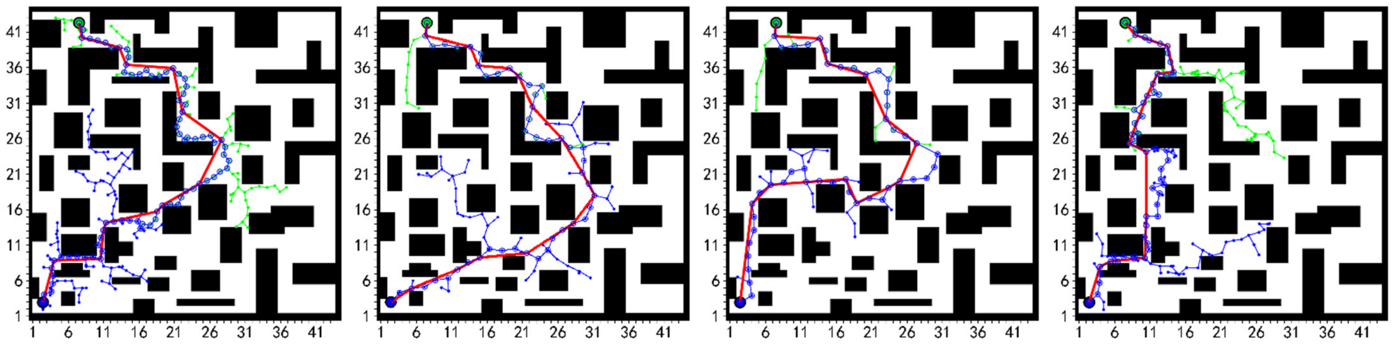

(**e**) (**f**) (**g**) (**h**)

**Figure S1.** Comparative path planning results for Instance P1. (**a**) Proposed algorithm. (**b**) Single offline PSO-optimized Bi-RRT. (**c**) Improved Bi-RRT without PSO. (**d**) RRT* algorithm. (**e**) RRT-Connect algorithm. (**f**) Bi-RRT algorithm. (**g**) APF-Bi-RRT algorithm. (**h**) GWO-Bi-RRT algorithm.

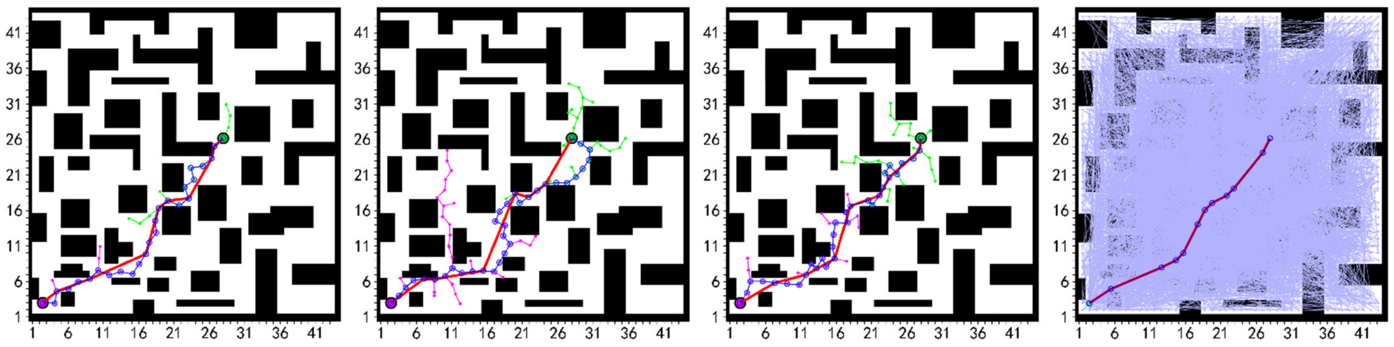

(**a**) (**b**) (**c**) (**d**)

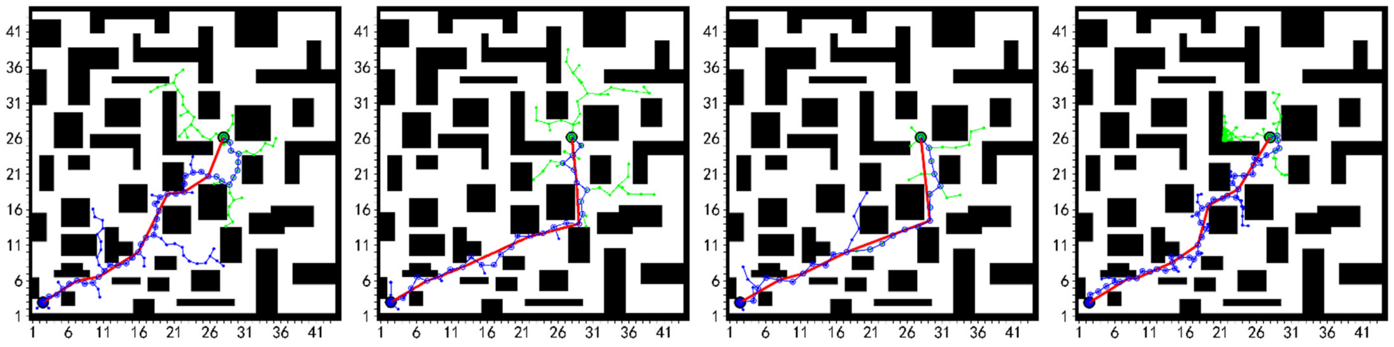

(**e**) (**f**) (**g**) (**h**)

**Figure S2.** Comparative path planning results for Instance P2. (a) Proposed algorithm. (b) Single offline PSO-optimized Bi-RRT. (c) Improved Bi-RRT without PSO. (d) RRT* algorithm. (e) RRT-Connect algorithm. (f) Bi-RRT algorithm. (g) APF-Bi-RRT algorithm. (h) GWO-Bi-RRT algorithm.

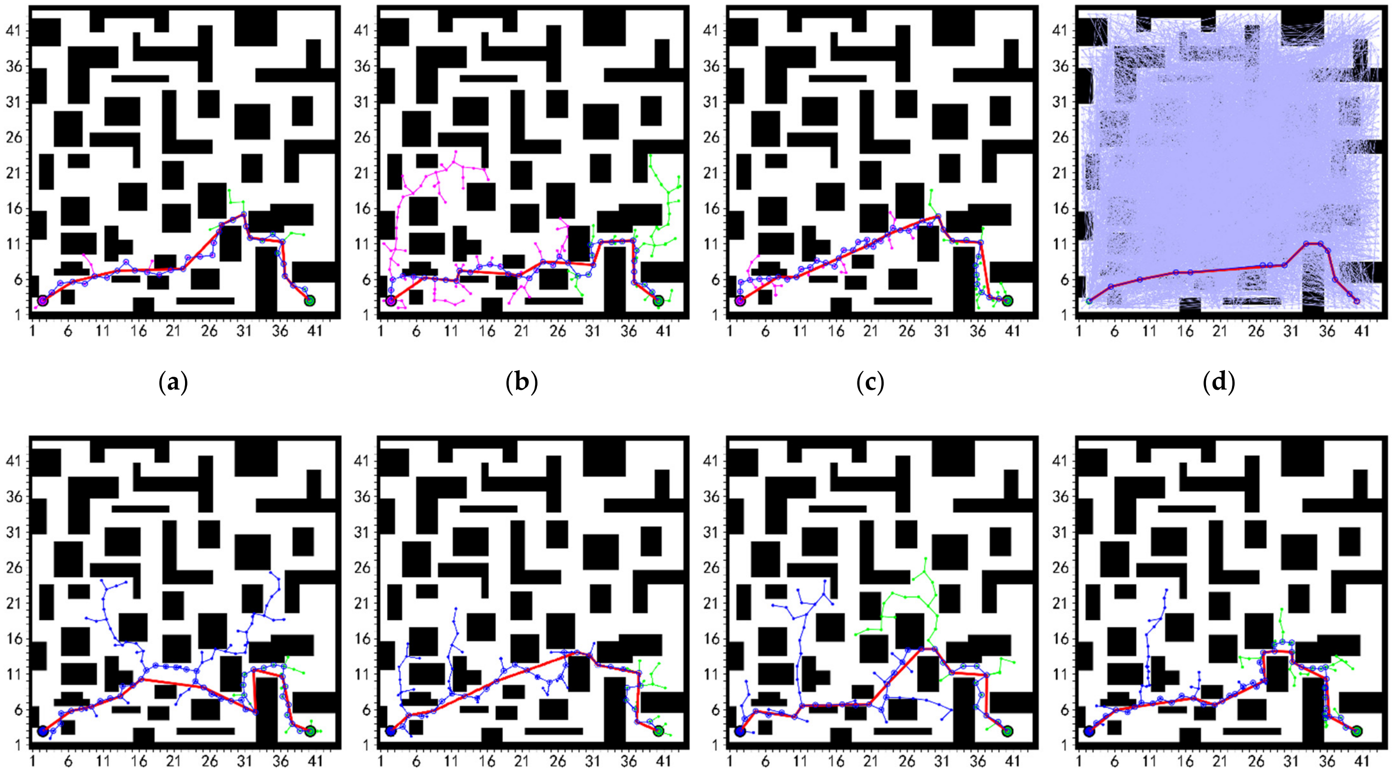

(a) (b) (c) (d) (e) (f) (g) (h)

**Figure S3.** Comparative path planning results for Instance P3. (a) Proposed algorithm. (b) Single offline PSO-optimized Bi-RRT. (c) Improved Bi-RRT without PSO. (d) RRT* algorithm. (e) RRT-Connect algorithm. (f) Bi-RRT algorithm. (g) APF-Bi-RRT algorithm. (h) GWO-Bi-RRT algorithm.

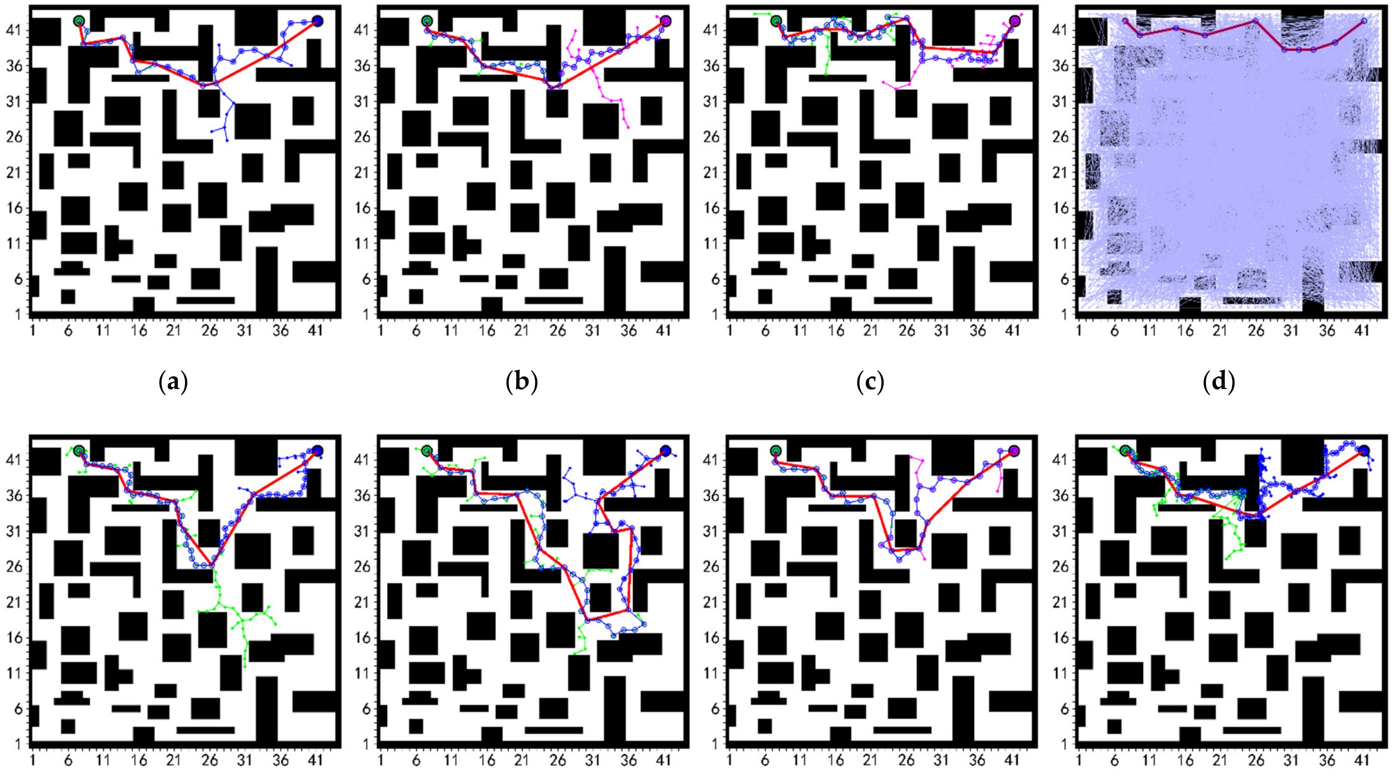

(a) (b) (c) (d) (e) (f) (g) (h)

**Figure S4.** Comparative path planning results for Instance P4. (a) Proposed algorithm. (b) Single offline PSO-optimized Bi-RRT. (c) Improved Bi-RRT without PSO. (d) RRT* algorithm. (e) RRT-Connect algorithm. (f) Bi-RRT algorithm. (g) APF-Bi-RRT algorithm. (h) GWO-Bi-RRT algorithm.

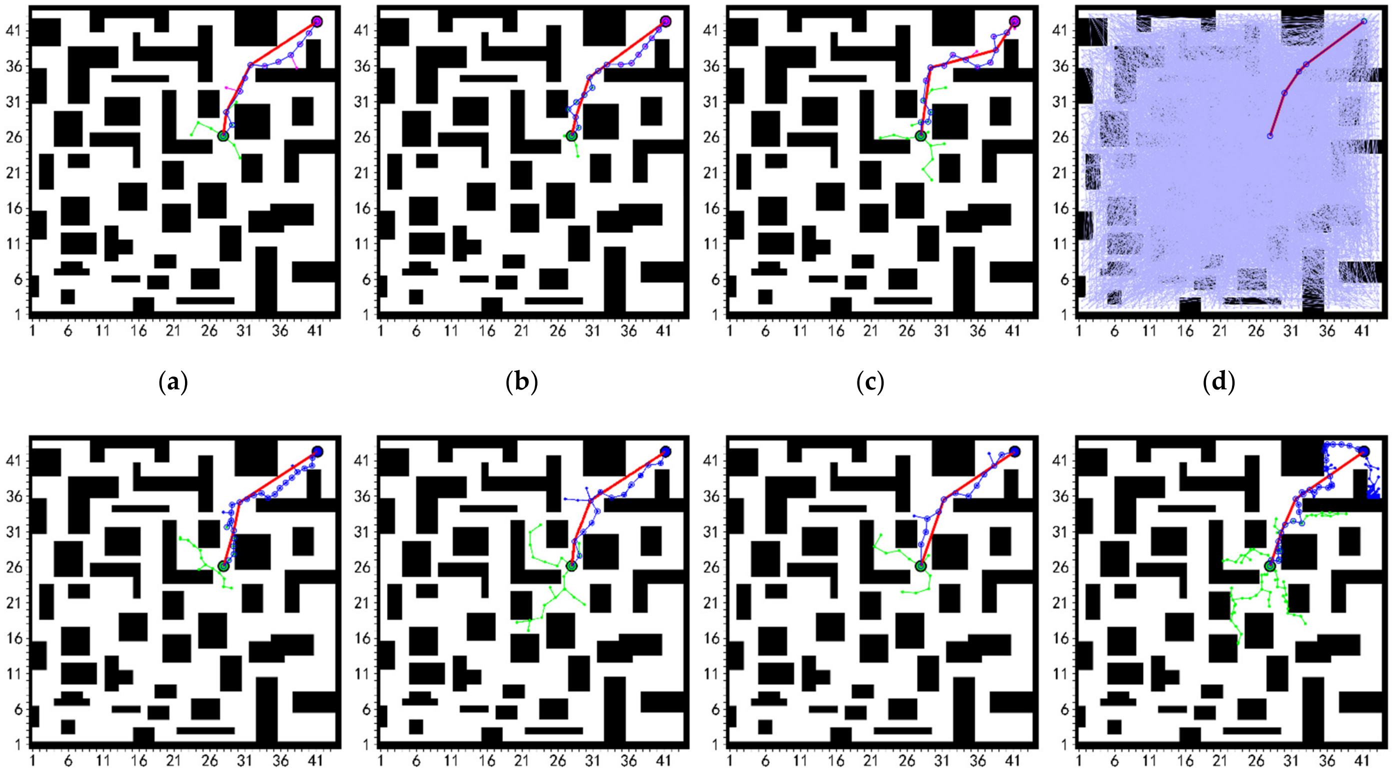


(a) (b) (c) (d)

(e) (f) (g) (h)

**Figure S5.** Comparative path planning results for Instance P5. (a) Proposed algorithm. (b) Single offline PSO-optimized Bi-RRT. (c) Improved Bi-RRT without PSO. (d) RRT* algorithm. (e) RRT-Connect algorithm. (f) Bi-RRT algorithm. (g) APF-Bi-RRT algorithm. (h) GWO-Bi-RRT algorithm.

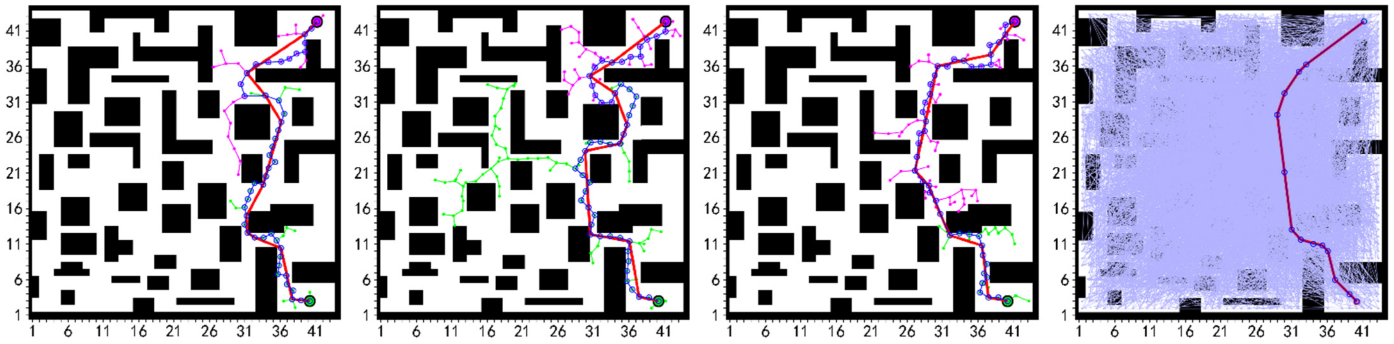


(a) (b) (c) (d)

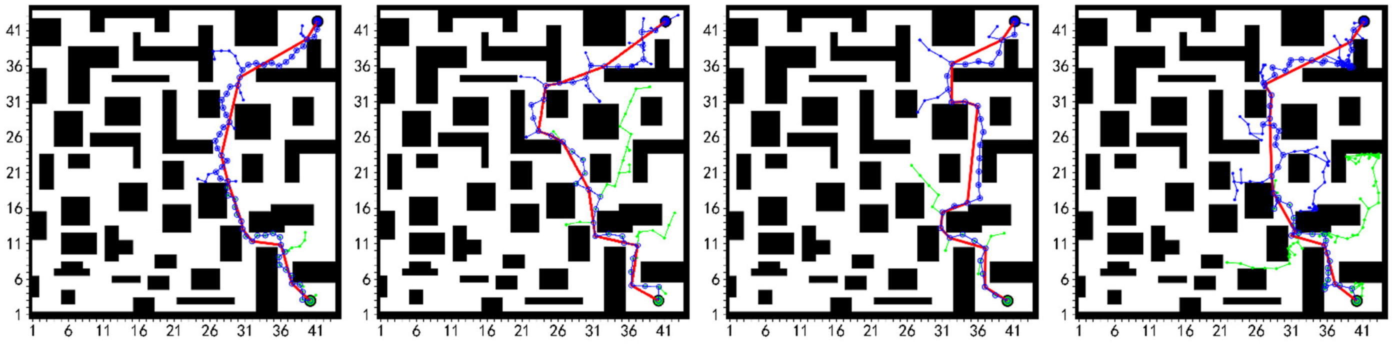

(**e**) (**f**) (**g**) (**h**)

**Figure S6.** Comparative path planning results for Instance P6. (a) Proposed algorithm. (b) Single offline PSO-optimized Bi-RRT. (c) Improved Bi-RRT without PSO. (d) RRT* algorithm. (e) RRT-Connect algorithm. (f) Bi-RRT algorithm. (g) APF-Bi-RRT algorithm. (h) GWO-Bi-RRT algorithm.

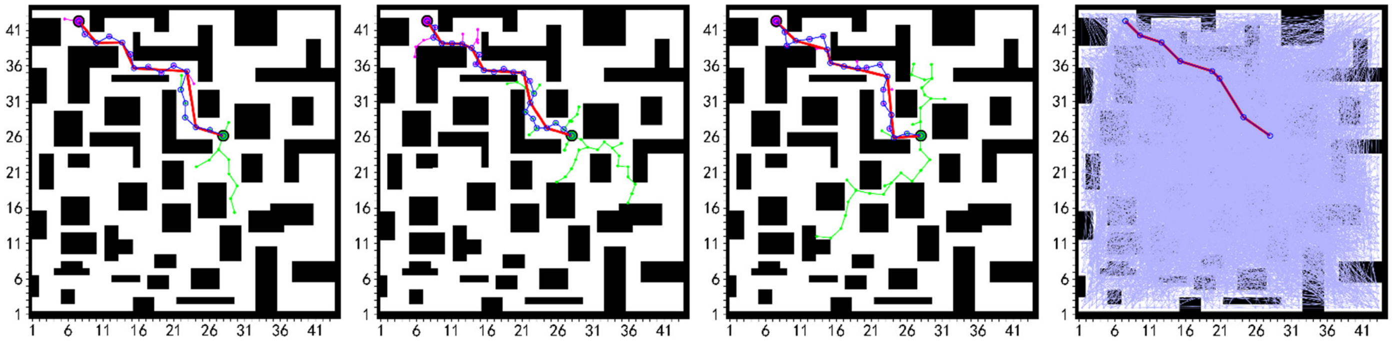

(**a**) (**b**) (**c**) (**d**)

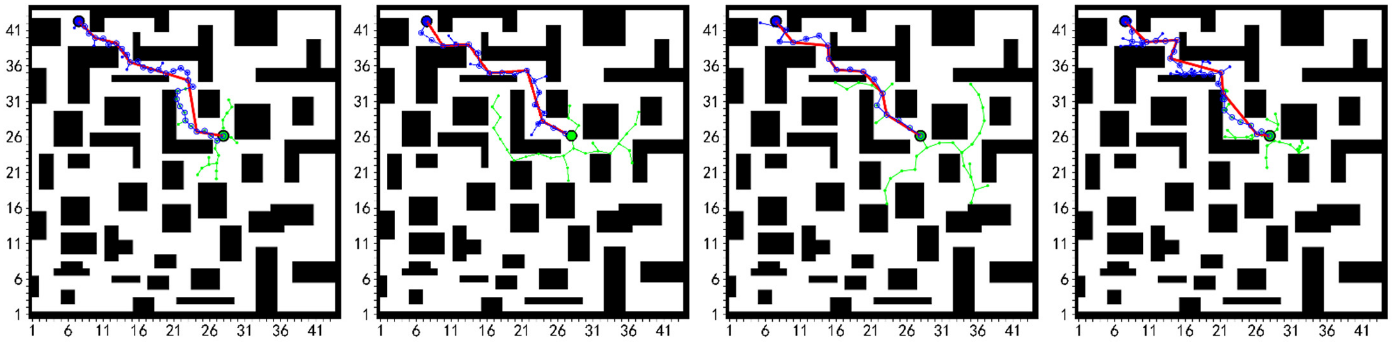

(**e**) (**f**) (**g**) (**h**)

**Figure S7.** Comparative path planning results for Instance P7. (a) Proposed algorithm. (b) Single offline PSO-optimized Bi-RRT. (c) Improved Bi-RRT without PSO. (d) RRT* algorithm. (e) RRT-Connect algorithm. (f) Bi-RRT algorithm. (g) APF-Bi-RRT algorithm. (h) GWO-Bi-RRT algorithm.

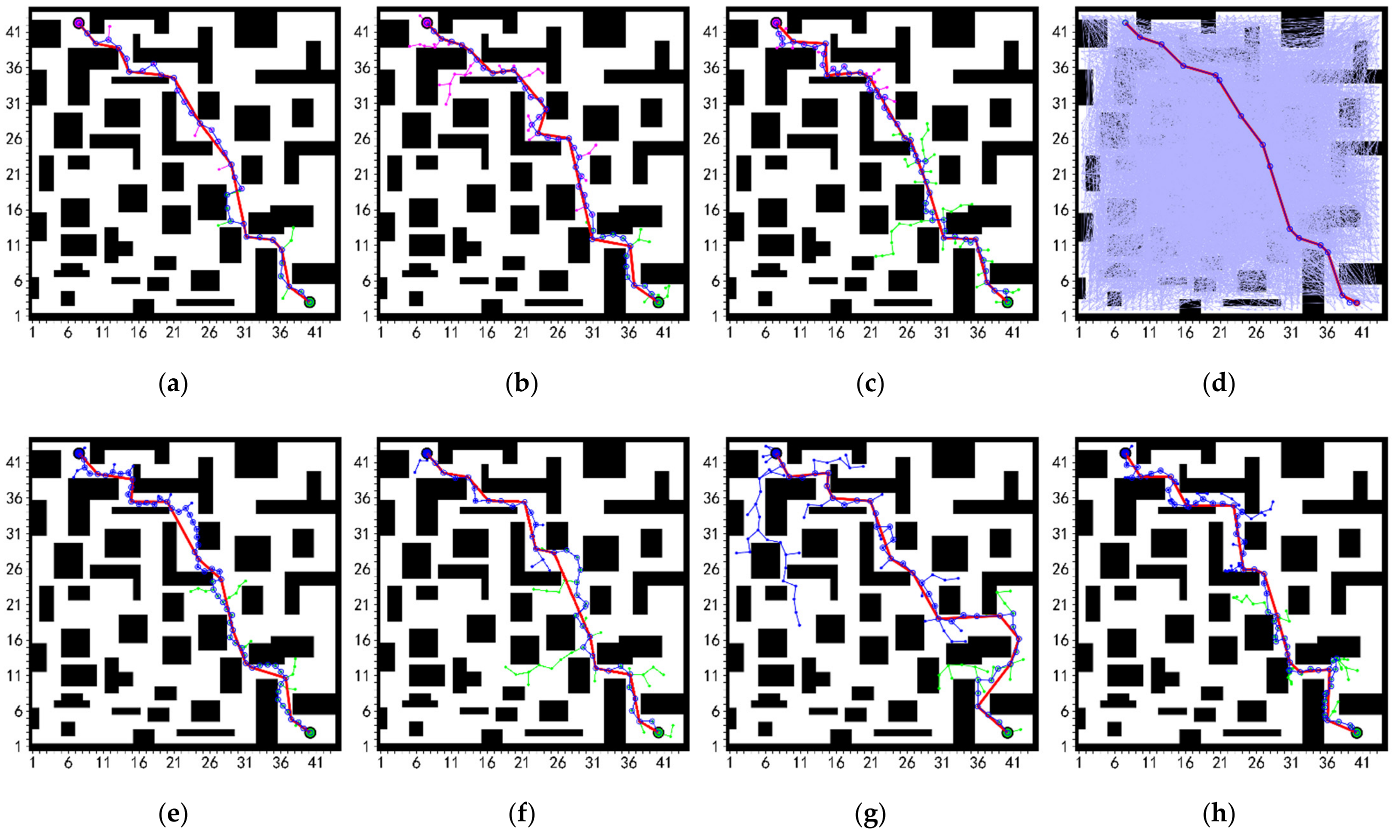


**Figure S8.** Comparative path planning results for Instance P8. (a) Proposed algorithm. (b) Single offline PSO-optimized Bi-RRT. (c) Improved Bi-RRT without PSO. (d) RRT* algorithm. (e) RRT-Connect algorithm. (f) Bi-RRT algorithm. (g) APF-Bi-RRT algorithm. (h) GWO-Bi-RRT algorithm.

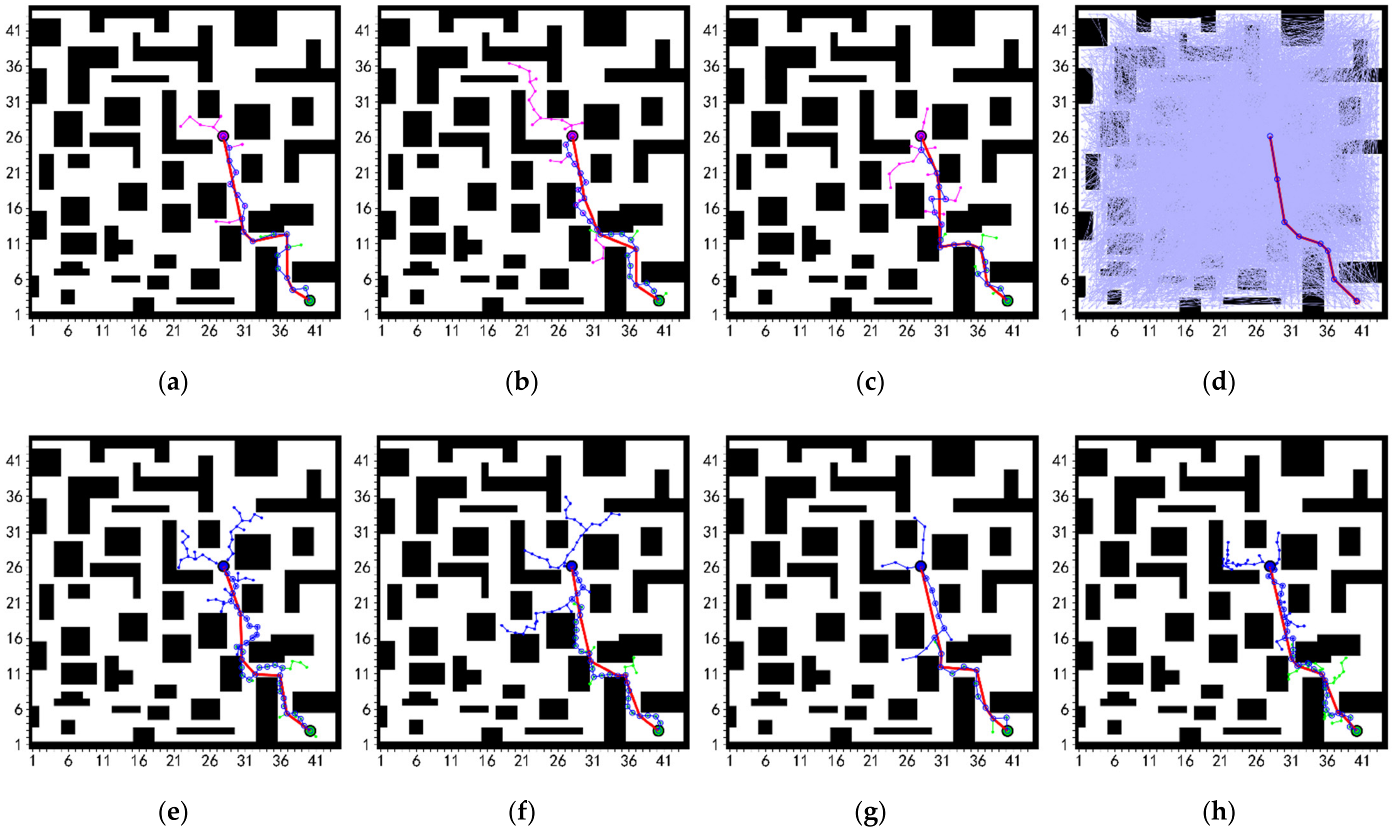

**Figure S9.** Comparative path planning results for Instance P9. (a) Proposed algorithm. (b) Single offline PSO-optimized Bi-RRT. (c) Improved Bi-RRT without PSO. (d) RRT* algorithm. (e) RRT-Connect algorithm. (f) Bi-RRT algorithm. (g) APF-Bi-RRT algorithm. (h) GWO-Bi-RRT algorithm.
